# MicroRNAs and Metabolites in Serum Change after Chemotherapy: Impact on Hematopoietic Stem and Progenitor Cells

**DOI:** 10.1371/journal.pone.0128231

**Published:** 2015-05-29

**Authors:** Thomas Walenda, Yvonne Diener, Edgar Jost, Elizabeth Morin-Kensicki, Tamme W. Goecke, Andreas Bosio, Björn Rath, Tim H. Brümmendorf, Ute Bissels, Wolfgang Wagner

**Affiliations:** 1 Helmholtz Institute for Biomedical Engineering, RWTH Aachen University Medical School, Aachen, Germany; 2 Miltenyi Biotec GmbH, Bergisch Gladbach, Germany; 3 Department for Hematology, Oncology, Hemostaseology and Stem Cell Transplantation, RWTH Aachen University Medical School, Aachen, Germany; 4 Metabolon, Inc., Durham, NC, 27519, United States of America; 5 Department of Obstetrics and Gynecology, RWTH Aachen University Medical School, Aachen, German; 6 Department for Orthopedics, RWTH Aachen University Medical School, Aachen, Germany; German Red Cross Blood Service Frankfurt, GERMANY

## Abstract

Hematopoietic regeneration after high dose chemotherapy necessitates activation of the stem cell pool. There is evidence that serum taken after chemotherapy comprises factors stimulating proliferation and self-renewal of CD34^+^ hematopoietic stem and progenitor cells (HSPCs) – however, the nature of these feedback signals is yet unclear. Here, we addressed the question if specific microRNAs (miRNAs) or metabolites are affected after high dose chemotherapy. Serum taken from the same patients before and after chemotherapy was supplemented for *in vitro* cultivation of HSPCs. Serum taken after chemotherapy significantly enhanced HSPC proliferation, better maintained a CD34^+^ immunophenotype, and stimulated colony forming units. Microarray analysis revealed that 23 miRNAs changed in serum after chemotherapy – particularly, miRNA-320c and miRNA-1275 were down-regulated whereas miRNA-3663-3p was up-regulated. miRNA-320c was exemplarily inhibited by an antagomiR, which seemed to increase proliferation. Metabolomic profiling demonstrated that 44 metabolites were less abundant, whereas three (including 2-hydroxybutyrate and taurocholenate sulphate) increased in serum upon chemotherapy. Nine of these metabolites were subsequently tested for effects on HSPCs *in vitro*, but none of them exerted a clear concentration dependent effect on proliferation, immunophenotype and colony forming unit formation. Taken together, serum profiles of miRNAs and metabolites changed after chemotherapy. Rather than individually, these factors may act in concert to recruit HSPCs into action for hematopoietic regeneration.

## Introduction

Under steady state conditions, hematopoietic stem and progenitor cells (HSPCs) reside quiescent in their bone marrow niche [1]. However, they possess the potential to greatly increase proliferation and self-renewal if required—e.g. after high dose chemotherapy followed by hematopoietic stem cell transplantation (HSCT) [2]. Hematopoietic reconstitution after allogeneic or autologous HSCT is usually observed within weeks, but it is still unclear which signals recruit the HSPCs into action [3]. In a previous study, we demonstrated that systemically released factors in patient serum after HSCT contribute to this regulatory process [4]. We identified growth factors which change in serum upon chemotherapy, e.g. three isoforms of platelet-derived growth factor (PDGF), leukemia inhibitory factor (LIF), chemokine [C-X-C motif] ligand 10 (IP-10) and chemokine [C-C motif] ligand 2 (MCP-1), but none of them seemed to be relevant as a unique signaling factor. Despite the common perception that growth factors play a leading role in hematopoietic regulation, there might be other mechanisms involved such as microRNAs (miRNAs) or metabolites.

MicroRNAs are short, non-coding RNAs that regulate gene expression at the post-transcriptional level by binding to target mRNAs and thereby either inhibiting their translation or accelerating their degradation [5]. Beside their intracellular localization, miRNAs have recently been found to circulate in blood and various other body fluids and their expression changes according to physiological and pathological conditions [6,7]. It is conceivable that such circulating miRNAs are actively secreted to mediate signaling effects. Little is known about the origin of miRNAs circulating in serum, but they may be predominantly secreted by blood cells [8]. To prevent RNA degradation and to target miRNAs to the recipient, miRNAs can be packed into microvesicles, such as exosomes [9], or they can be associated with high-density lipoproteins [10]. These microvesicles or lipoprotein-miRNA complexes have been shown to be taken up by recipient cells via endocytosis—and hence circulating miRNAs can influence recipient cells and modify their behavior. MicroRNAs are master regulators of cellular development. They regulate cell fate decisions such as lineage commitment and differentiation but also self-renewal of HSPCs [11,12]. It has even been shown that modulation of certain miRNAs promotes *in vitro* expansion of HSPCs [13–15] or at least maintenance of a more primitive immunophenotype during *in vitro* cultivation [16]. Therefore, it is conceivable that specific miRNAs contribute to activation of the stem cell pool after high dose chemotherapy and HSCT.

Alternatively, metabolites might be relevant for regulation of stem cell function. They are intermediates and products of metabolism of usually less than 1 kDa in size. Recently, it has been shown that the niche regulates self-renewal of HSPCs via retinoic acid signaling [17]. Furthermore, there are studies indicating that HSPC quiescence is tightly regulated by the metabolic microenvironment [18,19]. Chemotherapy induces metabolic changes such as down-regulation of extracellular glutathione peroxidase and up-regulation of gamma-tocopherol concentration in patient serum [20]. Metabolomics—the quantitative analysis of metabolite profiles e.g. by mass-spectrometry—is ideally suited to identify relevant factors and this has been used for various cancer types. For example, metabolomics of colorectal cancer patients led to identification of circulating metabolites with significant changes in liver-only metastases and with extrahepatic metastases [21]. Other metabolites can be used as potential biomarker to predict response to neoadjuvant chemotherapy in breast cancer patients [22]. Furthermore, certain metabolites can influence the expression of miRNAs [23] and *vice versa*, and it has been shown that miRNAs can regulate metabolic pathways [24,25].

So far, it is still unclear if specific miRNAs or metabolites are relevant for activation of the hematopoietic stem cell pool. To this end, we performed a comparative analysis of patient serum before and after chemotherapy with particular focus on miRNAs and metabolites.

## Material and Methods

### Serum samples in the course of autologous HSCT

Ten mL of peripheral blood were collected from patients with lymphoma, acute myeloid leukemia (AML), multiple myeloma (MM) and healthy controls after written consent as described before [4]. Isolation of serum samples and the study were specifically approved by the Ethic Committee of RWTH Aachen University (Permit Number: EK155/09). Blood samples taken before chemotherapy (BC) and during leukopenia after HSCT (AC) were transferred into a 15 mL tube (Greiner, Kremsmünster, Austria), agitated horizontally at 37°C for 1 h to allow coagulation, then incubated upright at 4°C for 4 h and finally centrifuged at 840 x g for 15 min [26]. Serum supernatant was aliquoted and stored at -80°C until use. Further information on serum samples and patients is provided in Tables A, B, C and D in S1 File.

### Isolation of hematopoietic stem and progenitor cells and of mesenchymal stromal cells

CD34^+^ cells were isolated from fresh umbilical cord blood after written consent. Isolation of CD34^+^ cells and the study were specifically approved by the Ethic Committee of RWTH Aachen University (Permit Number: EK187/08). For antagomiR experiments we used CD133^+^ cells, which were isolated from cord blood from DKMS Nabelschnurblutbank (Dresden, Germany). Isolation of CD133^+^ cells from fresh cord blood (DKMS Nabelschnurblutbank, Dresden, Germany) and the study were specifically approved by the local Ethics Committee of the Ärztekammer Nordrhein (Permit Number: EK103/2011). In brief, mononuclear cells were separated by density gradient centrifugation and CD34^+^ or CD133^+^ cells were enriched using MicroBead Kits according to the manufacturer’s instructions (Miltenyi Biotec GmbH, Bergisch Gladbach, Germany) [27]. For co-culture conditions, MSCs were isolated from the *caput femoris* after the patient’s written consent and cultivated as described before [4,28]. Isolation of MSCs from bone marrow and the study were specifically approved by the Ethic Committee of RWTH Aachen University (Permit Number: EK128/09). MSCs were seeded as feeder cells between passages 3 to 6 (10 to 15 population doublings).

### Culture conditions and expansion of HSPCs with serum supplementation

Hematopoietic stem and progenitor cells were expanded in 24-well plates (Nunc) in StemSpan serum-free expansion medium (Stem Cell Technologies, Grenoble, France) either without stromal support or upon co-culture on a confluent layer of MSCs. Culture medium was supplemented in parallel with 10% of each serum sample (BC or AC) [4]. In order not to falsify potential serum effects on HSPCs, no further cytokines were supplemented to the culture medium.

### Analysis of cell division history

Freshly isolated HSPCs were labeled with carboxyfluorescein diacetate N-succinimidyl ester (CFSE; Sigma-Aldrich) or the CellTrace Violet Cell Proliferation Kit (Violet Dye, Life Technologies, Carlsbad, CA, 92008, USA) to monitor cell divisions [27]. The fluorescent dye thereby binds to protein residues resulting in a homogenously stained cytoplasm. The fluorescent dye is then equally distributed to the daughter cells within each cell division (higher proliferation entails lower fluorescence intensity). In brief, cells were washed in PBS and then stained with CFSE at a final concentration of 2.5 μM in PBS with 0.1% fetal calf serum (FCS; PAA Laboratories, Cölbe, Germany) for 10 min at 37°C. Violet Dye was used at a final concentration of 1.67 μM in PBS. The staining reaction was stopped with ice cold PBS (PAA) with 10% FCS for 5 min on ice followed by three washing steps with PBS. HSPCs were then expanded and after four to seven days; CFSE or Violet Dye intensity was measured together with immunophenotype by flow cytometry using a FACS Canto II (BD) or a MACSQuant Analyzer 10 (Miltenyi Biotec).

### Immunophenotypic analysis

CD34^+^ cells were washed in PBS, stained with CD34-allophycocyanin (APC; Becton Dickinson, San Jose, CA, USA [BD], clone 8G12), CD38-PE (BD, clone HB-7) and CD45-V500 (BD, clone HI30) in a dilution of 1:200 and analyzed using a FACS Canto II (BD) running FACS Diva software (BD). Further analysis was performed using WinMDI software (WinMDI 2.8; The Scripps Institute, San Diego, CA, USA). Discrimination between MSCs and HSPCs was possible by forward scatter, side scatter, propidium iodide (PI) staining and CFSE-staining. CD133^+^ cells, which were used for antagomiR experiments, were stained with CD133/2-PE (clone 293C3) and CD34-APC (clone AC136) according to the manufacturer’s protocol (Miltenyi Biotec). Analysis was performed with the MACSQuant Analyzer 10 and the MACSQuant Software (Miltenyi Biotec).

### miRNA profiling

Frozen serum samples were thawed at room temperature. Total RNA was extracted from 500 μL of serum using TRIzol LS Reagent (Life technologies, Darmstadt, Germany) and the miRNeasy mini kit (Qiagen, Hilden, Germany) according to the manufacturer’s instructions. The RNA sample volume was reduced in a Speed Vac to a final volume of 1 μL and labeled and hybridized using the Human microRNA Microarray Kit (Rel16.0, Agilent Technologies, Santa Clara, CA, USA) according to the manufacturer’s protocol. The detailed hybridization protocol and raw data are provided in NCBI's Gene Expression Omnibus (GEO, Series accession number GSE57570). For statistical analysis, signal intensities were normalized to the array median, log2 transformed and subjected to Student’s paired t-test. For heat map presentation, the Multiple Experiment Viewer tool version 4.9.0 (Dana-Farber Cancer Institute, Boston, MA, USA) was used. Prediction of miRNA targets was performed using TargetScan [29]. The predicted targets were classified in Gene Ontology categories of molecular function to estimate relevant biological processes. As background for the analysis, the probe set of the Agilent Whole Human Genome Oligo Microarray (8x60k) was chosen. Term enrichment relative to the expected background distribution was scored using Fisher’s Exact test with Benjamini–Hochberg correction.

### qRT-PCR of miRNAs

Total RNA was extracted from 500 μL of serum, reduced to a final volume of 6 μL and subsequently used for reverse transcription applying the miScript II RT Kit (Qiagen). For Real-Time PCR, the miScript SYBR Green PCR Kit and miScript Primer Assay (Hs_miR-320_3) were used according to the manufacturer’s protocol (miScript PCR System, Qiagen). PCR was performed in an ABI 7900HT Fast Real-Time PCR System (Life technologies).

### Inhibition of miRNA-320c activity in HSPCs

For inhibition of miRNA-320c activity, we used cholesterol-modified antagomiRs targeting miRNA-320c (anta-320c, ACCCUCUCAACCCAGCUUUU) or a non-targeting control (anta-Neg). AntagomiRs were synthesized by Miltenyi Biotec according to Krützfeld et al. [30]. CD133^+^ cells were stained with Violet Dye, cultivated overnight and then electroporated with antagomiRs using the CD34 Cell Nucleofector Kit and Nucleofector II device from Amaxa (Lonza, Cologne, Germany) according to the manufacturer’s protocol. We used 7 to 9 x 10^4^ cells per sample and a final antagomiR concentration of 3 μM. Electroporation efficiency was assessed using 2 μM of non-targeting siRNA labeled with AlexaFluor 488 (AF488-siRNA) or 2.4 μg of a GFP-encoding mRNA. After electroporation, cells were cultured overnight in 500 μL StemSpan serum-free expansion medium supplemented with 10 ng/mL human stem cell factor (SCF), 20 ng/mL human thrombopoietin (TPO), 10 ng/mL human fibroblast growth factor 1 (FGF-1, all from Miltenyi Biotec), 10 μg/mL heparin (Ratiopharm GmbH, Ulm, Germany) and penicillin / streptomycin (PAA) and then re-seeded in 96-well U-bottom plates (BD) at 5 x 10^3^ cells per well in 150 μL StemSpan serum-free expansion medium supplemented with 10% human AB-serum (Dunn Labortechnik GmbH, Asbach, Germany) without further cytokines. Violet Dye intensity and immunophenotype were analyzed by flow cytometry (MACSQuant Analyzer 10) four days after electroporation.

### Metabolomics

Biomarkers in serum samples were analyzed in cooperation with Metabolon Inc. (Durham, NC, USA). In brief, biochemical profiling was performed using multiple platform mass spectrometry technology consisting of liquid chromatography/mass spectrometry (LC/MS), gas chromatography/mass spectrometry (GC/MS) and accurate mass determination and MS/MS fragmentation (LC/MS) to cover a broad profile of 297 small molecule metabolites. Analysis and interpretation of data was performed by Metabolon Inc. Metabolites were identified by automated comparison and spectra fitting to a chemical standard library of experimentally derived spectra as previously described [31–33]. Missing values were imputed by the lowest measured value to avoid bias from different detection levels. Data were then median normalized, log2 transformed and subjected to paired two-sited Student’s t-test analysis. Further analysis and heatmap presentation was performed with the TIGR MeV tool (Dana-Farber Cancer Institute, Boston, MA, USA).

### Supplementation of metabolites

Hematopoietic stem and progenitor cells were expanded in 24-well plates (Nunc) in StemSpan serum free expansion medium (Stem Cell Technologies, Grenoble, France) either without stromal support or on a confluent layer of MSCs as described above. Culture medium was supplemented with 10 ng/mL stem cell factor (SCF; PeproTech GmbH, Hamburg, Germany), 20 ng/mL thrombopoietin (TPO; PeproTech), 10 ng/mL fibroblast growth factor 1 (FGF-1; PeproTech) and 10 μg/mL heparin (Roche GmbH, Mannheim, Germany) as previously described [28]. Furthermore, metabolites were added in a range of different concentrations that in most cases presumably cover physiological concentrations: gamma-tocopherol (Sigma Aldrich; 10 μM to 1 mM; physiological concentration: 0–40 μM, [34]), lactate (Roth; 1–100 μM; physiological concentrations: 0.3–1.3 μM [35]), alanine (Roth; 1–100 μM), uridine (Roth; 1–100 μM; physiological concentrations: 5.2 μM [36]), taurocholic acid sodium salt hydrate (Sigma Aldrich; 1–100 μM; total bile acid concentration range: 3.75–4.17 μM [37]), L-glutamic acid monosodium salt hydrate (Sigma Aldrich; 1–100 mM), N^G^,N^G^-dimethylarginine dihydrochloride (Sigma Aldrich; 1–100 μM; physiological concentrations: 6,8 μM [38]), tert-butyl(R)-2-hydroxybutyrate (Sigma Aldrich; 10 μM—1 mM; physiological concentrations: 31.3 μM [39]) and 12(S)-Hydroxy-(5Z,8Z,10E,14Z)-eicosatetraenoic acid (Sigma Aldrich; 10 nM—1 μM) were supplemented to the culture medium as indicated in the text.

### Colony forming unit assay

Colony forming unit (CFU) potential was determined to estimate expansion of HSPCs with serum before and after chemotherapy as we have described before [4] and to estimate culture expansion of HSPCs upon treatment with metabolites or antagomiRs. 12,500 CD34^+^ cells were grown in StemSpan medium supplemented with SCF, TPO, FGF-1 and heparin as described above. Metabolites were added in the highest concentration of the previous tests. AntagomiR-electroporated cells were cultured as described above. After seven days, the progeny of HSPCs was re-seeded in different dilutions (1:10, 1:100 and 1:1000 dilution) in 24-well culture dishes with 500 μL methylcellulose medium per well (HSC-CFU lite with EPO; Miltenyi Biotec). After two additional weeks of incubation with methylcellulose medium, granulocyte (CFU-G), macrophage (CFU-M) and erythrocyte colonies (CFU-E) were counted according to the manufacturer’s instructions.

### Statistical analysis

Unless stated otherwise we adopted the Student’s t-test (paired analysis if applicable) to estimate statistical significance. A P-value of either ≤ 0.05 or ≤ 0.01 was considered as statistically significant (as indicated in the text).

## Results

### Serum after chemotherapy stimulates proliferation of HSPCs

**Fig 1:**
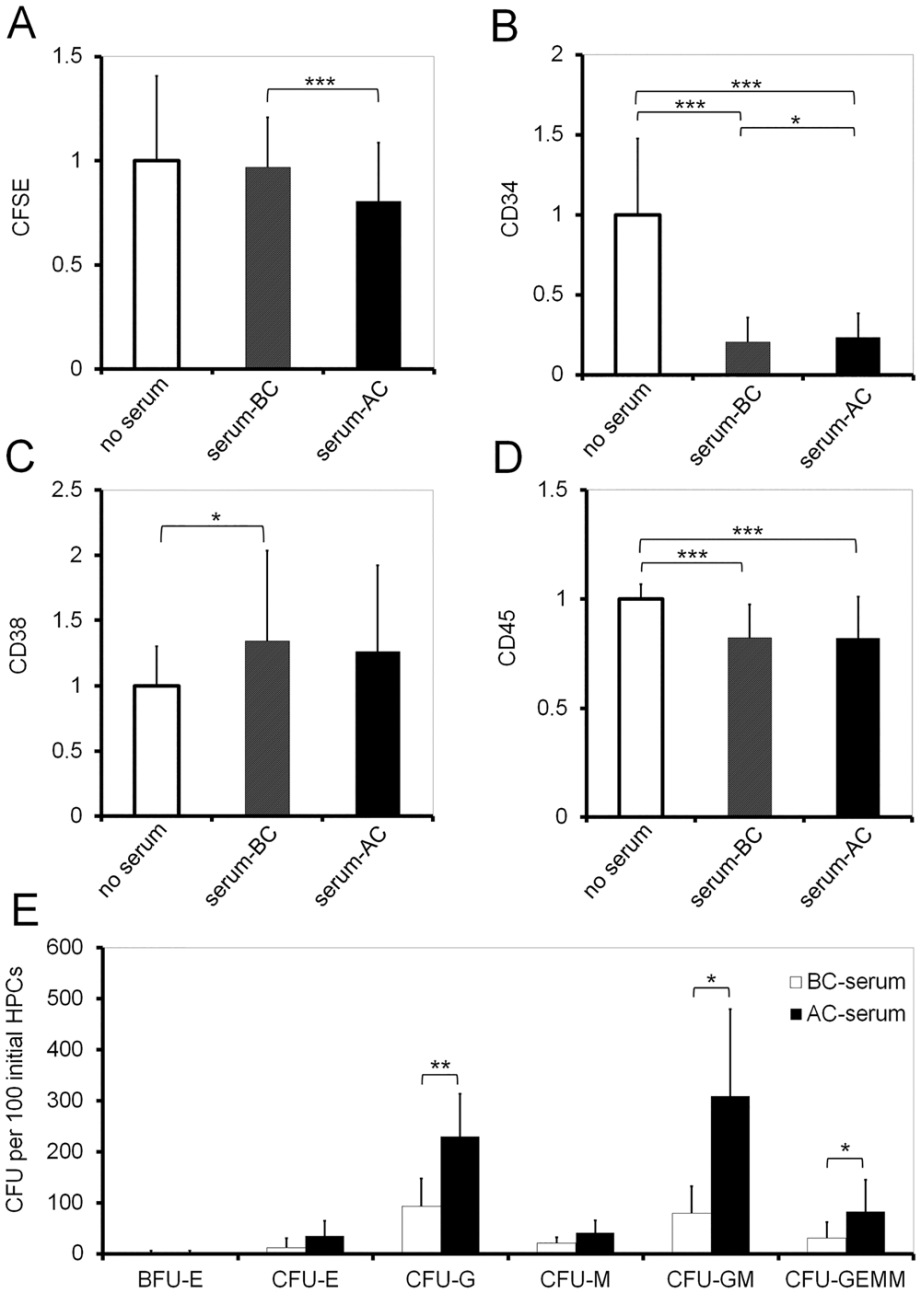
Impact of serum on HSPC proliferation, immunophenotype, and CFU potential. HSPCs were stained with CFSE and *in vitro* cultured for five days with 10% serum supplementation. Serum was derived from patients before (BC) and after (AC) chemotherapy. As control (Ctr.) cells were cultured without serum. (A) CFSE mean intensity (lower signal intensity indicates higher proliferation rate), as well as mean fluorescence intensities of (B) CD34, (C) CD38, and (D) CD45 were then determined via flow cytometry (intensities were normalized to the corresponding control; n = 5). (E) HSPCs were cultured with BC or AC serum for seven days and were then re-seeded in methylcellulose medium. After two weeks numbers of burst forming unit erythrocyte (BFU-E), colony forming unit erythrocyte (CFU-E), granulocyte (CFU-G), macrophage (CFU-M), granulocyte and macrophage (CFU-GM) as well as granulocyte, erythrocyte, megakaryocyte and macrophage (CFU-GEMM) were counted. (n = 2). *P < 0.05; **P < 0.01; Error bars represent SD.

Serum was taken from ten patients before chemotherapy (BC) and after chemotherapy (AC, see Table A in S1 File). These serum samples were then supplemented in parallel to culture media for *in vitro* expansion of HSPCs (always 10% serum). CD34^+^ cells from umbilical cord blood were stained with CFSE to monitor cell division. After five days of culture, flow cytometric analysis revealed that serum samples taken during neutropenia (AC) enhanced proliferation of HSPCs significantly as compared to corresponding samples taken before chemotherapy (BC) (Fig 1A; five independent experiments, each of them with serum samples of all 10 patients; P = 2.74 x 10^–8^). This growth-promoting effect was not observed under co-culture with mesenchymal stromal cells (MSCs) which might be attributed to the massive growth-promoting effect of the supportive cellular environment (Fig. A in S1 File). Furthermore, serum after chemotherapy maintained higher levels of CD34 as determined by flow cytometry (Fig 1B)—despite the fact that increased proliferation is usually associated with loss of CD34 [27]. The same effect was also observed under co-culture conditions with MSCs (Fig. A in S1 File). Notably, serum-free controls revealed much higher CD34 expression than serum BC or AC and this might also be attributed to lower proliferation rates. No significant differences were detected in serum BC *versus* AC with regard to CD38 and CD45 expression either with or without co-culture with MSCs even though there were some changes as compared to controls without serum additives (Fig 1C and 1D; and Fig. A in S1 File). Subsequently, we tested if blood parameters—such as cell counts of leucocytes, thrombocytes, erythrocytes and hemoglobin—correlate with *in vitro* proliferation of HSPC. This analysis was performed separately for BC serum (Fig. B in S1 File) and AC serum samples (Fig. B in S1 File). After chemotherapy, particularly serum of anemic patients significantly stimulated HSPC proliferation as compared to non-anemic samples (erythrocyte count: P = 0.046, hemoglobin level: P = 0.009, Pearson correlation). Subsequently, we determined CFU potential as a surrogate assay to estimate expansion of HSPCs under the influence of either BC or AC serum. CD34^+^ cells were cultured for seven days with 10% serum supplements and then re-seeded in methylcellulose medium for two additional weeks. Overall, formation of CFU-G, CFU-GM and CFU-GEMM was significantly higher in AC serum (Fig 1E) [4].

### Differentially expressed miRNAs in serum samples before and after chemotherapy

**Fig 2:**
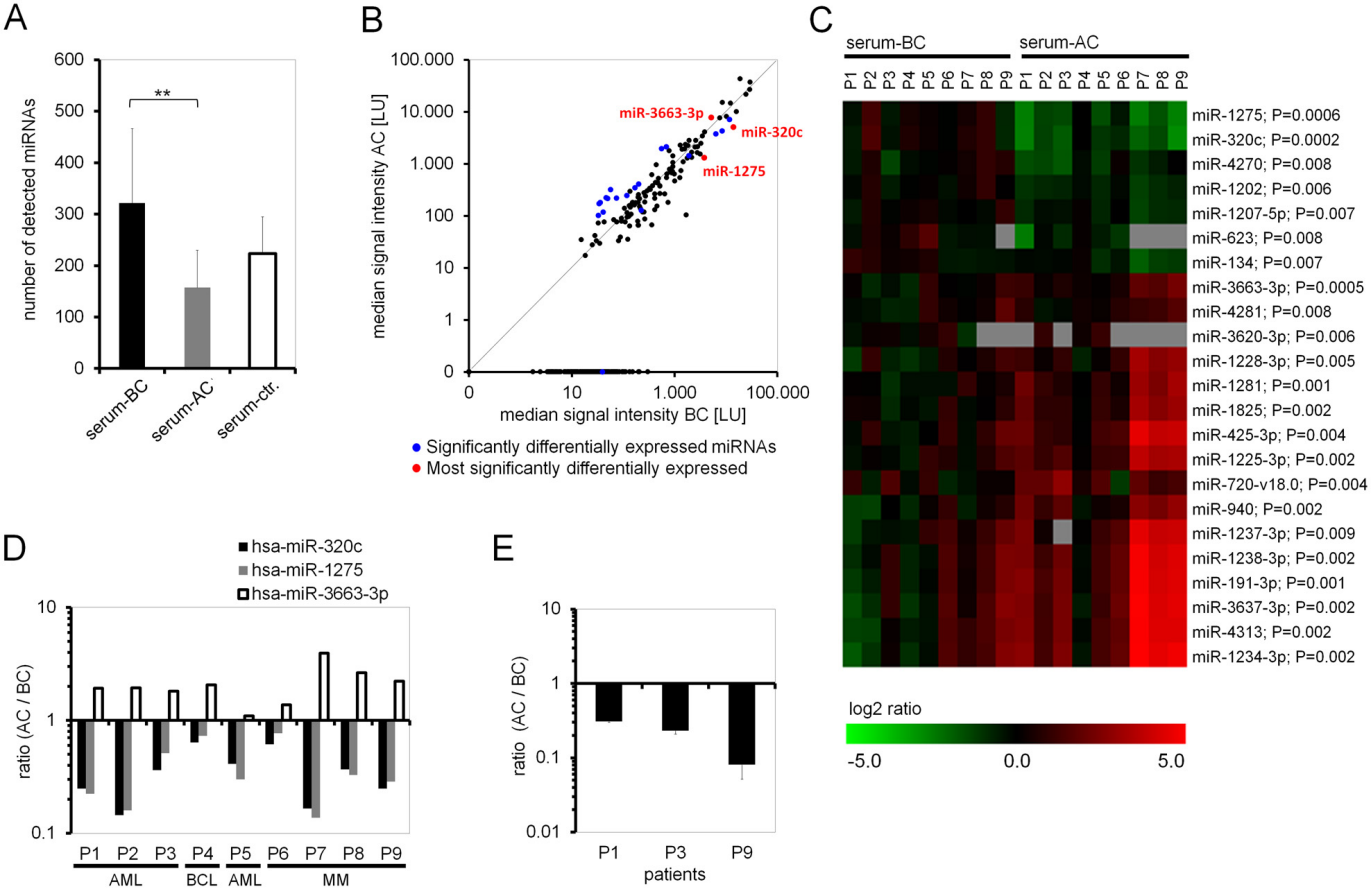
Differential miRNA expression in serum before and after chemotherapy. (A) Bar chart displaying the average number of detected miRNAs in serum samples of patients before chemotherapy (BC; n = 9), after chemotherapy (AC; n = 9), and of healthy controls (n = 7). Error bars represent SD, **P = 0.004. (B) Median signal intensities AC *versus* BC (n = 9). Significantly differentially expressed microRNAs after normalization are marked in blue; the three most significant miRNAs miRNA-320c, miRNA-1275 and miRNA-3663-3p are marked in red. Data points on the x-axis represent 163 miRNAs that are exclusively detected in BC serum. No miRNAs were exclusively detected in AC serum. (C) Heat map of significantly differentially expressed miRNAs in patient samples BC and AC (P ≤ 0.01, paired t-test). Displayed is the log2-transformed ratio of the normalized signal intensities *versus* median signal intensity of BC serum. (D) The three most significantly differentially expressed miRNAs showed consistent expression patterns in serum of all analyzed patients (Ratio of normalized signal intensities in serum AC *versus* BC). (E) qRT-PCR confirmed increased expression of miRNA-320c in serum BC compared to AC. Fold change of miRNA expression is shown as mean of triplicates for three patient sample pairs. Error bars represent SD.

MicroRNAs might be relevant for the stimulatory effect of serum after chemotherapy, and therefore we compared miRNA profiles in serum samples from nine patients before and after therapy (Table B in S1 File). Overall, the number of detected miRNAs decreased significantly in serum taken after chemotherapy (Fig 2A) and signal intensities decreased as compared to serum before chemotherapy (Fig 2B). The global miRNA expression level correlated significantly with leucocyte numbers (Fig. C in S1 File; P = 0.0004), and this was also true for known hematopoietic miRNAs such as miRNA-486-5p, miRNA-22 and miRNA-150 [40,41]. This may indicate that most of the observed changes in miRNA expression are due to neutropenia after HSCT. Taking this into consideration, the microarray data were normalized to the array median. Using paired t-test, we found 23 miRNAs to be significantly differentially expressed in BC serum *versus* AC serum (P ≤ 0.01, Fig 2C). MicroRNA-320c (P = 0.0002), miRNA-1275 (P = 0.0005), and miRNA-3663-3p (P = 0.0006) revealed the most significant differences. MicroRNA-320c and miRNA-1275 were increased in samples before chemotherapy, whereas miRNA-3663-3p was higher after chemotherapy (Fig 2D). These miRNAs were also amongst the most significantly differentially expressed genes when we compared AC *versus* BC serum samples separately for AML and MM patients, indicating that these changes seem to be independent from disease and treatment (Fig. D in S1 File). Furthermore, the results were exemplarily validated by quantitative real-time PCR (qRT-PCR) of miRNA-320c (Fig 2E)—a miRNA that is predicted to target a broad range of developmentally relevant genes (Fig. E in S1 File).

### Inhibition of miRNA-320c

Circulating miRNAs can be taken up by recipient cells [8] and therefore we reasoned that decreased serum levels of miRNAs may impact on hematopoietic regeneration. To further address the functional relevance of specific miRNAs, we exemplarily silenced miRNA-320c in HSPCs. Since miRNA-320c is down-regulated in AC serum, we hypothesized that hematopoiesis supportive effects might be mimicked by electroporation with the corresponding antagomiR (anta-320c). AntagomiRs are chemically engineered oligonucleotides that efficiently and persistently silence endogenous miRNAs [42]. Electroporation of anta-320c resulted in a significantly increased proliferation of HSPCs as compared to control cells (anta-Neg) (Fig. F in S1 File). However, CD34 expression decreased upon treatment with anta-320c and the number of CFUs was not significantly affected (Fig. F in S1 File). Efficiency of electroporation was determined in previous optimization experiments (Fig. F in S1 File): using a fluorescently labeled, non-targeting siRNA (AF488-siRNA) or a GFP mRNA, we obtained 90.3 and 96.8% of fluorescent cells, respectively, indicating that the delivery of RNA molecules was highly efficient—and therefore we expect that the observed effects of anta-320c are due to knockdown of the corresponding miRNA. Thus, inhibition of miRNA-320c is not sufficient to mimic the hematopoiesis supportive effects of serum taken under neutropenia.

### Metabolomic changes after chemotherapy

**Fig 3:**
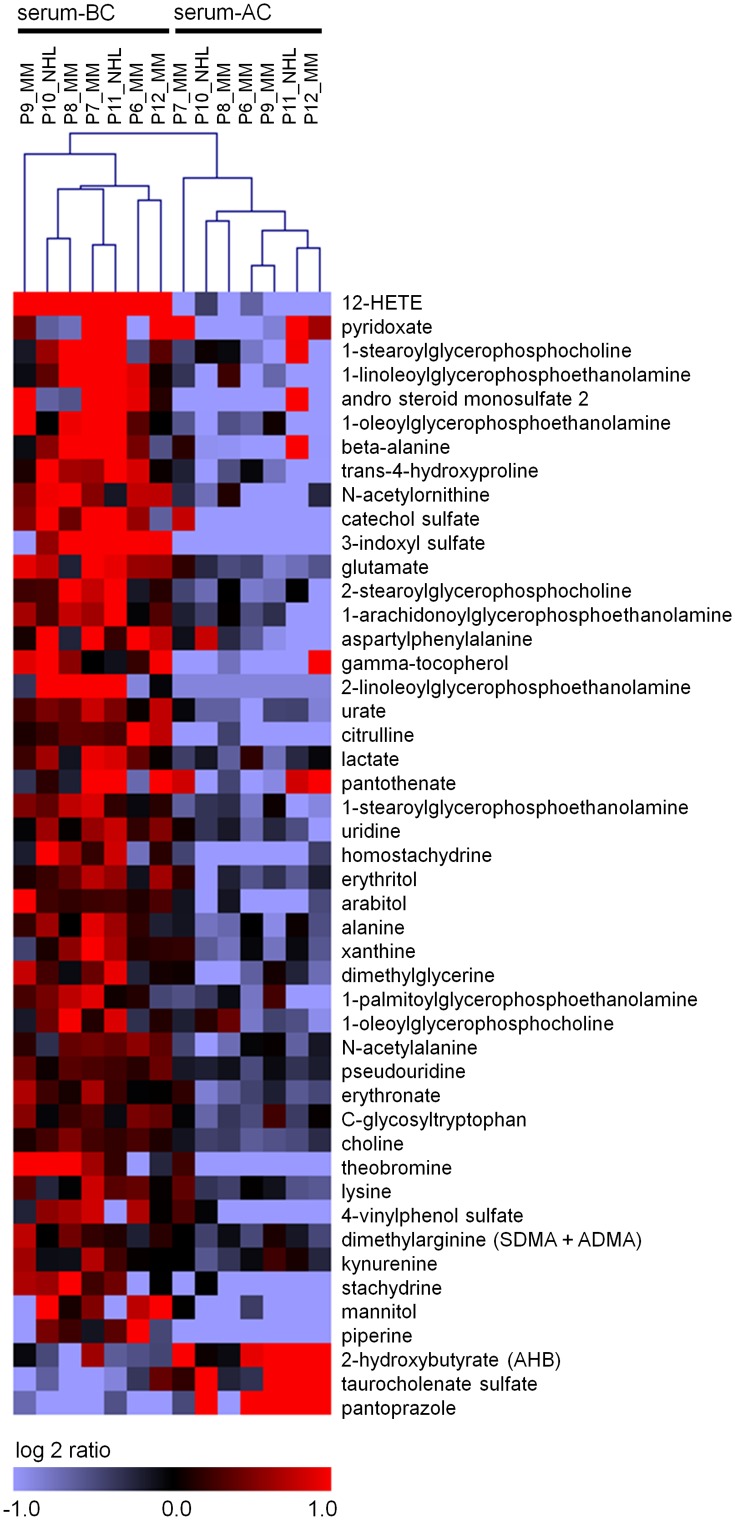
Heat map of significantly differentially detected metabolites. Seven samples before (BC) and after chemotherapy (AC) were analyzed with regard to their metabolite composition. 44 metabolites were significantly down-regulated and three were up-regulated after chemotherapy (Multiple Myeloma (MM), non-Hodgekin Lymphoma follicular (NHL); P ≤ 0.01; paired t-test).

Subsequently, we analyzed metabolite profiles in serum BC *versus* AC using multiple mass spectrometry technology. The identified dataset comprises a total of 297 named biochemicals. Upon log transformation and imputation with minimum observed values for each compound, 47 metabolites differed significantly between serum BC and AC (Fig 3; n = 7; paired t-test; P ≤ 0.01; and Table C in S1 File): 44 metabolites were less abundant after chemotherapy such as citrulline, urea cycle components, proline, 4-hydroxyproline, the dipeptide proline-hydroxyproline, choline, glycerol-3-phosphate, glycerophosphorylcholine many lysolipids, an oxidized form of arachidonic acid (12-HETE), and theobromine. Only three metabolites revealed higher abundance upon chemotherapy: 2-hydroxybutyrate, taurocholenate sulfate and pantoprazole. Overall, none of the tested metabolites clearly correlated with cell counts (leukocytes, thrombocytes, erythrocytes, and hemoglobin; Figs. G, H, I and J in S1 File) – except for uridine, which moderately increases with leucocyte numbers (P = 0.038, Pearson rank correlation)—indicating that changes in metabolites not solely attributed to the changing composition of blood cells. In fact, pantoprazole was given to the patients as a stomach protection after chemotherapy whereas lower concentration of theobromine is related to changes in the patient’s nutrition upon HSCT. This exemplifies that there are significant changes in metabolite profiles of serum after chemotherapy which can be successfully tracked by the metabolomics approach.

### Functional testing of various metabolites on HSPC *in vitro* expansion

**Fig 4:**
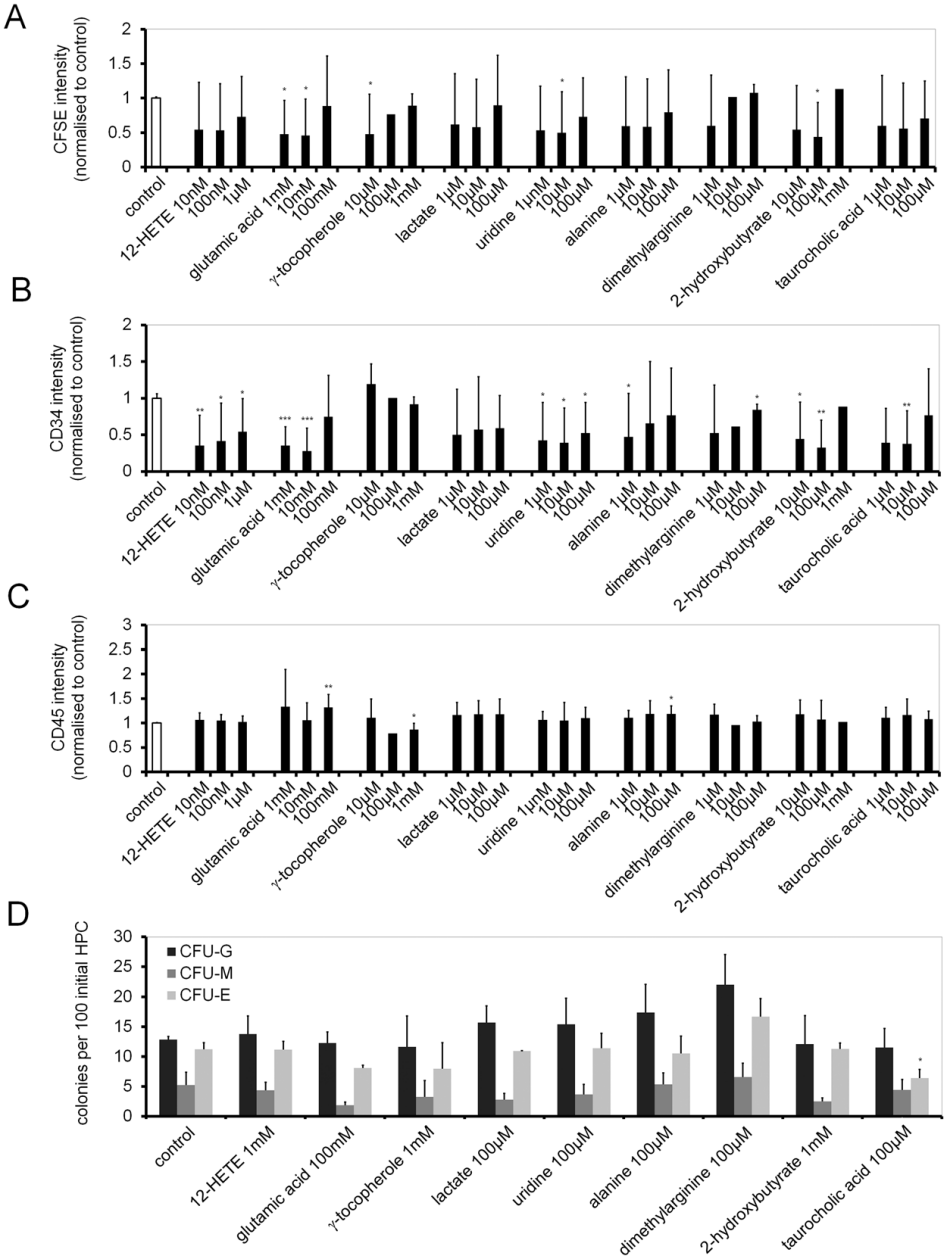
Proliferation and immunophenotypic analysis upon addition of metabolites. HSPCs were stained with CFSE and *in vitro* cultured for five days with supplementation of metabolites. These experiments were performed in parallel using three different concentrations for each metabolite as indicated. (A) CFSE intensity as well as (B) expression of CD34 and (C) CD45 were then determined via flow cytometry and normalized to the corresponding control. Error bars represent SD. (D) Alternatively, HSPCs were cultured for seven days in parallel with each of the metabolites and then reseeded in methylcellulose medium. After two additional weeks the numbers of erythrocyte (CFU-E), granulocyte (CFU-G) and macrophage (CFU-M) colonies were counted. Error bars represent SEM, *P ≤ 0.05, **P ≤ 0.01, ***P ≤ 0.001; n = 3.

To further analyze the functional relevance of specific metabolites, we tested their effect on *in vitro* culture of HSPCs. CD34^+^ HSPCs were isolated from CB, stained with CFSE and cultured either with or without stromal support. Culture medium was supplemented with 12-HETE, glutamic acid, gamma-tocopherol, lactate, uridine, alanine, dimethylarginine, 2-hydroxybutyrate and taurocholic acid in three concentrations that were expected to cover the physiological concentrations (Fig 4A). After five days, cells were analyzed by FACS and residual CFSE-staining as well as expression of CD34 and CD45 were determined. Several metabolites increased proliferation particularly at lower concentrations, but these results hardly reached statistical relevance. Loss of CD34 expression correlated with faster cell proliferation. For example, addition of 1 mM or 10 mM glutamic acid lead to significantly reduced CFSE intensity and expression of CD34 compared to control samples without metabolite addition (Fig 4A–4C). In contrast, CD45 expression was hardly influenced. Even in co-culture with MSCs the metabolites hardly influenced proliferation and surface marker expression (Fig. K in S1 File).

Subsequently, we determined colony forming unit (CFU) potential as a surrogate assay to estimate expansion of HSPCs. CD34^+^ cells were cultured for seven days with each of the nine metabolites. Cells were then reseeded in methylcellulose medium, and after 12 to 14 days granulocyte (CFU-G), macrophage (CFU-M) and erythrocyte colonies (CFU-E) were counted. Addition of 100 μM taurocholic acid significantly reduced CFU-E rate compared to controls without addition of metabolites, whereas no significant impact on colony formation rate of the other eight tested metabolites was detected (Fig 4D).

## Discussion

Hematopoietic stress after chemotherapy necessitates tight regulation of self-renewal and differentiation of HSPCs. Our results support the notion that this is associated with systemically released factors, which are also active under *in vitro* culture conditions. In this study, we considered patients of different disease background and therapeutic regimen—e.g. myeloid leukemia patients undergoing cytarabine based consolidation *versus* multiple myeloma patients treated with high-dose melphalan therapy and autologous HSCT. Such parameters are certainly relevant confounders, which need to be further addressed by larger datasets. Further relevant factors may be parenteral nutrition and the consecutive inflammatory milieu. However, our results indicate similar hematopoiesis supportive activity, and similar changes in miRNA and metabolites were observed in our clinically heterogeneous patient cohort. Notably, most of the miRNAs and metabolites which revealed significant changes upon chemotherapy were down-regulated. Initially, we anticipated that increased self-renewal is associated with up-regulation of specific factors. Alternatively, it is also conceivable that stem cell function is suppressed under steady state conditions by specific molecules which are down-regulated during hematopoietic stress.

Recently, we have developed mathematical models for hematopoietic regeneration upon autologous stem cell transplantation [43,44] and for disease development of myelodysplastic syndromes (MDS) [45]. All of these studies postulated feedback signals acting on proliferation and differentiation of the stem cell pool, and all of them were in line with experimental validation using serum samples for *in vitro* culture of HSPCs. Thus, there is evidence that factors involved in this regulatory mechanism are not only acting locally in the bone marrow niche, but also at systemic level, but however, the questions remain which molecules govern this process.

Our current study provides evidence that the miRNA-landscape changes in the course of chemotherapy. Overall, the miRNA-levels decline in AC serum. This may partly be attributed to reduction of disease-specific miRNAs which have been described in various malignant disorders including AML, MM [46], and acute lymphoblastic leukemia (ALL) [47]. This might also explain why in tendency miRNA-levels in serum BC were higher than in healthy controls. However, there is evidence that most of the circulating miRNAs originate from blood cells and that perturbations in blood cell counts can alter the miRNA expression profile in serum [48]. This is in line with our observed correlation of leucocyte counts and global miRNA levels including typical hematopoietic miRNAs such as miRNA-486-5p. Therefore, we had to normalize miRNA profiles to identify specific miRNAs with significant changes. Down-regulation of miRNA-320c and miRNA-1275 after chemotherapy might indicate that these miRNAs have a negative effect on proliferation.

Recently, it has been demonstrated that the miRNA-320 family suppresses stem cell-like characteristics in prostate cancer cells by down-regulating the Wnt/beta-catenin signaling pathway [49]—a pathway which is also of central importance for regulation of self-renewal and proliferation in hematopoietic stem cells [50,51]. Furthermore, miRNA-320 targets the transferrin receptor CD71 in the human leukemia cell line HL-60, thereby inhibiting proliferation [52]. We could show that inhibition of miRNA-320c activity using an antagomiR has a growth-promoting effect on HSPCs—yet the mean CD34 expression decreased as compared to controls. Therefore, it appears to be rather unlikely that down-regulation of miRNA-320c is the only relevant factor for increased proliferation of HSPCs because many other miRNAs were also changing in AC serum. If down-regulation of these miRNAs is functionally relevant, then they probably act in concert.

Beside changes in the miRNA landscape, we identified 47 metabolites that were significantly changed in AC serum, and again most of them were down-regulated. Down-regulation of proline, 4-hydroxyproline and proline-hydroxyproline may reflect changes in tissue and extracellular matrix remodeling [53,54]. Furthermore, decrease of choline, glycerol-3-phosphate, glycerophosphorylcholine, lysolipids, and of the arachidonic acid oxidation product 12-HETE may indicate decreased phospholipid turnover after chemotherapy [55]. AC serum showed significantly reduced levels gamma-tocopherol, a form of vitamin E. Moreover, it has also been described that gamma-tocopherol increases prior to HSCT [20]. Incubation of HSPCs with 10 μM gamma-tocopherol slightly increased proliferation of HSPCs, although it has been suggested that gamma-tocopherol inhibits cell cycle progression in cancer by down-regulation of cyclins [56]. Thus, these effects appear to be specific for culture conditions and cell types. 2-Hydroxybutyrate was found to be an early biomarker of both insulin resistance and impaired glucose regulation [57]. Elevated concentrations of bile acids like taurocholic acid impair proliferation and increase apoptosis in Jurkat cells [58]. Our results with specific metabolites did not reveal consistent and pronounced effects on proliferation, immunophenotype or CFU-potential of HSPCs. In fact, several results partly contradict each other—e.g. taurocholic acid, which was increased after chemotherapy in patient serum, moderately reduced the CFU-E rate, although higher proliferation was observed with serum of rather anemic patients. Thus, none of the metabolites appears to be an exclusive regulator of hematopoiesis—probably a network of factors is needed to recapitulate the hematopoiesis supportive effects of serum during aplasia.

Until now, research has mainly focused on characterization of HSPCs and methods for their *in vitro* expansion to obtain sufficient cell numbers for HSCT [59]. Mechanisms for the activation of stem cell function in the course of chemotherapy have hardly been addressed. Therefore, a better understanding of the physiologic mechanisms that recruit the stem cell pool into action may provide new perspectives for regenerative medicine without requirement of transplantation. Our study demonstrates that aplasia after high dose chemotherapy is also associated with significant changes in miRNA and metabolite profiles. Most of them were less abundant after chemotherapy and there was no clear evidence that one of them is playing a unique role for activation of the stem cell pool. It is however conceivable that these factors act in concert with other regulatory factors. The hematopoietic stem cell niche provides a very complex environment which is modulated by growth factors, miRNAs, and metabolites—even though so far none of them seems to govern hematopoiesis *per se*.

## Supporting Information

S1 File Combined pdf with Figs. A—K and Tables A—D. Fig. A, Impact of serum on HSPC proliferation and immunophenotype during co-culture with MSCs. Fig. B, Correlation of HSPC proliferation with blood parameters of serum samples. Fig. C, Detected miRNA numbers correlate with leukocyte count. Fig. D, Differential miRNA expression in serum before and after chemotherapy among patient subgroups. Fig. E, Gene ontology analysis of miRNA-320c targets. Fig. F, Inhibition of miRNA-320c activity enhances HSPC proliferation. Fig. G, Correlation of metabolite levels with patient’s leukocyte count. Fig. H, Correlation of metabolite levels with patient’s thrombocyte count. Fig. I, Correlation of metabolite levels with patient’s erythrocyte count. Fig. J, Correlation of metabolite levels with patient’s hemoglobin concentration. Fig. K, Effects of metabolites on HSPCs in co-culture with MSCs. Table A, Serum samples used as cell culture supplement. Table B, Serum samples used for miRNA profiling. Table C, Serum samples used for metabolomic profiling. Table D, Detailed patient treatment information.
(PDF)

## References

[1] Wilson A, Oser GM, Jaworski M, Blanco-Bose WE, Laurenti E, Adolphe C et al Dormant and self-renewing hematopoietic stem cells and their niches. Ann N Y Acad Sci 2007; 1106:64–75. 17442778 10.1196/annals.1392.021

[2] Brown JA, Boussiotis VA. Umbilical cord blood transplantation: basic biology and clinical challenges to immune reconstitution. Clin Immunol 2008;127(3):286–297. 10.1016/j.clim.2008.02.008 18395491 PMC2468219

[3] Spiegel A, Kalinkovich A, Shivtiel S, Kollet O, Lapidot T. Stem cell regulation via dynamic interactions of the nervous and immune systems with the microenvironment. Cell Stem Cell 2008;3(5):484–492. 10.1016/j.stem.2008.10.006 18983964

[4] Walenda T, Bokermann G, Jost E, Galm O, Schellenberg A, Koch CM et al Serum after autologous transplantation stimulates proliferation and expansion of human hematopoietic progenitor cells. PLoS ONE 2011;6(3): e18012 10.1371/journal.pone.0018012 21437259 PMC3060918

[5] Bartel DP. MicroRNAs: genomics, biogenesis, mechanism, and function. Cell 2004;116(2):281–297. 14744438 10.1016/s0092-8674(04)00045-5

[6] Keller A, Leidinger P, Bauer A, Elsharawy A, Haas J, Backes C et al Toward the blood-borne miRNome of human diseases. Nat Methods 2011;8(10):841–843. 10.1038/nmeth.1682 21892151

[7] Grasedieck S, Sorrentino A, Langer C, Buske C, Dohner H, Mertens D et al Circulating microRNAs in hematological diseases: principles, challenges, and perspectives. Blood 2013;121(25):4977–4984. 10.1182/blood-2013-01-480079 23550041

[8] Liang H, Gong F, Zhang S, Zhang CY, Zen K, Chen X. The origin, function, and diagnostic potential of extracellular microRNAs in human body fluids. Wiley Interdiscip Rev RNA 2014;5(2):285–300. 10.1002/wrna.1208 24259376

[9] Valadi H, Ekstrom K, Bossios A, Sjostrand M, Lee JJ, Lotvall JO. Exosome-mediated transfer of mRNAs and microRNAs is a novel mechanism of genetic exchange between cells. Nat Cell Biol 2007;9(6):654–659. 17486113 10.1038/ncb1596

[10] Vickers KC, Palmisano BT, Shoucri BM, Shamburek RD, Remaley AT. MicroRNAs are transported in plasma and delivered to recipient cells by high-density lipoproteins. Nat Cell Biol 2011;13(4):423–433. 10.1038/ncb2210 21423178 PMC3074610

[11] Bissels U, Bosio A, Wagner W. MicroRNAs are shaping the hematopoietic landscape. Haematologica 2012;97(2):160–167. 10.3324/haematol.2011.051730 22058204 PMC3269472

[12] Havelange V, Garzon R. MicroRNAs: emerging key regulators of hematopoiesis. Am J Hematol 2010;85(12):935–942. 10.1002/ajh.21863 20941782

[13] Guo S, Lu J, Schlanger R, Zhang H, Wang JY, Fox MC et al MicroRNA miR-125a controls hematopoietic stem cell number. Proc Natl Acad Sci U S A 2010;107(32):14229–14234. 10.1073/pnas.0913574107 20616003 PMC2922532

[14] Han YC, Park CY, Bhagat G, Zhang J, Wang Y, Fan JB et al microRNA-29a induces aberrant self-renewal capacity in hematopoietic progenitors, biased myeloid development, and acute myeloid leukemia. J Exp Med 2010;207(3):475–489. 10.1084/jem.20090831 20212066 PMC2839143

[15] Lechman ER, Gentner B, van GP, Giustacchini A, Saini M, Boccalatte FE et al Attenuation of miR-126 activity expands HSC in vivo without exhaustion. Cell Stem Cell 2012;11(6):799–811. 10.1016/j.stem.2012.09.001 23142521 PMC3517970

[16] Gerrits A, Walasek MA, Olthof S, Weersing E, Ritsema M, Zwart E et al Genetic screen identifies microRNA cluster 99b/let-7e/125a as a regulator of primitive hematopoietic cells. Blood 2012;119(2):377–387. 10.1182/blood-2011-01-331686 22123844

[17] Ghiaur G, Yegnasubramanian S, Perkins B, Gucwa JL, Gerber JM, Jones RJ. Regulation of human hematopoietic stem cell self-renewal by the microenvironment's control of retinoic acid signaling. Proc Natl Acad Sci U S A 2013;110(40):16121–16126. 10.1073/pnas.1305937110 24043786 PMC3791732

[18] Takubo K, Nagamatsu G, Kobayashi CI, Nakamura-Ishizu A, Kobayashi H, Ikeda E et al Regulation of glycolysis by Pdk functions as a metabolic checkpoint for cell cycle quiescence in hematopoietic stem cells. Cell Stem Cell 2013;12(1):49–61. 10.1016/j.stem.2012.10.011 23290136 PMC6592822

[19] Desplat V, Ivanovic Z, Dupuis F, Faucher JL, Denizot Y, Praloran V. Effects of lipoxygenase metabolites of arachidonic acid on the growth of human blood CD34(+) progenitors. Blood Cells Mol Dis 2000;26(5):427–436. 11112380 10.1006/bcmd.2000.0321

[20] White AC, Sousa AM, Blumberg J, Ryan HF, Fanburg BL, Kayyali US. Plasma antioxidants in subjects before hematopoietic stem cell transplantation. Bone Marrow Transplant 2006;38(7):513–520. 16980999 10.1038/sj.bmt.1705475

[21] Farshidfar F, Weljie AM, Kopciuk K, Buie WD, Maclean A, Dixon E et al Serum metabolomic profile as a means to distinguish stage of colorectal cancer. Genome Med 2012;4(5):42 10.1186/gm341 22583555 PMC3506908

[22] Wei S, Liu L, Zhang J, Bowers J, Gowda GA, Seeger H et al Metabolomics approach for predicting response to neoadjuvant chemotherapy for breast cancer. Mol Oncol 2013;7(3):297–307. 10.1016/j.molonc.2012.10.003 23142658 PMC5528483

[23] Giangreco AA, Nonn L. The sum of many small changes: microRNAs are specifically and potentially globally altered by vitamin D3 metabolites. J Steroid Biochem Mol Biol 2013;136:86–93. 10.1016/j.jsbmb.2013.01.001 23333596 PMC3686905

[24] Gao P, Tchernyshyov I, Chang TC, Lee YS, Kita K, Ochi T et al c-Myc suppression of miR-23a/b enhances mitochondrial glutaminase expression and glutamine metabolism. Nature 2009;458(7239):762–765. 10.1038/nature07823 19219026 PMC2729443

[25] Ito K, Suda T. Metabolic requirements for the maintenance of self-renewing stem cells. Nat Rev Mol Cell Biol 2014;15(4):243–256. 10.1038/nrm3772 24651542 PMC4095859

[26] Kurita M, iba-Kojima E, Shigeura T, Matsumoto D, Suga H, Inoue K et al Differential effects of three preparations of human serum on expansion of various types of human cells. Plast Reconstr Surg 2008;122(2):438–448. 10.1097/PRS.0b013e31817d618d 18626359

[27] Walenda T, Bork S, Horn P, Wein F, Saffrich R, Diehlmann A et al Co-Culture with Mesenchymal Stromal Cells Increases Proliferation and Maintenance of Hematopoietic Progenitor Cells. J Cell Mol Med 2010;14(1):337–350. 19432817 10.1111/j.1582-4934.2009.00776.x PMC3837622

[28] Walenda T*, Bokermann G*, Ventura Ferreira MS, Piroth DM, Hieronymus T, Neuss S et al Synergistic effects of growth factors and mesenchymal stromal cells for expansion of hematopoietic stem and progenitor cells. Exp Hematol 2011;36(4):617–628. 10.1016/j.exphem.2011.02.011 21356269

[29] Lewis BP, Burge CB, Bartel DP. Conserved seed pairing, often flanked by adenosines, indicates that thousands of human genes are microRNA targets. Cell 2005;120(1):15–20. 15652477 10.1016/j.cell.2004.12.035

[30] Krutzfeldt J, Rajewsky N, Braich R, Rajeev KG, Tuschl T, Manoharan M et al Silencing of microRNAs in vivo with 'antagomirs'. Nature 2005;438(7068):685–689. 16258535 10.1038/nature04303

[31] Lawton KA, Berger A, Mitchell M, Milgram KE, Evans AM, Guo L et al Analysis of the adult human plasma metabolome. Pharmacogenomics 2008;9(4):383–397. 10.2217/14622416.9.4.383 18384253

[32] Boudonck KJ, Mitchell MW, Nemet L, Keresztes L, Nyska A, Shinar D et al Discovery of metabolomics biomarkers for early detection of nephrotoxicity. Toxicol Pathol 2009;37(3):280–292. 10.1177/0192623309332992 19380839

[33] Evans AM, DeHaven CD, Barrett T, Mitchell M, Milgram E. Integrated, nontargeted ultrahigh performance liquid chromatography/electrospray ionization tandem mass spectrometry platform for the identification and relative quantification of the small-molecule complement of biological systems. Anal Chem 2009;81(16):6656–6667. 10.1021/ac901536h 19624122

[34] Wells SR, Jennings MH, Rome C, Hadjivassiliou V, Papas KA, Alexander JS. Alpha-, gamma- and delta-tocopherols reduce inflammatory angiogenesis in human microvascular endothelial cells. J Nutr Biochem 2010;21(7):589–597. 10.1016/j.jnutbio.2009.03.006 19443199

[35] Phypers B, Pierce T. Lactate physiology in health and disease. Contin Educ Anaesth Crit Care Pain 2006;6(3):128–132.

[36] Traut TW. Physiological concentrations of purines and pyrimidines. Mol Cell Biochem 1994;140(1):1–22. 7877593 10.1007/BF00928361

[37] Rainer PP, Primessnig U, Harenkamp S, Doleschal B, Wallner M, Fauler G et al Bile acids induce arrhythmias in human atrial myocardium—implications for altered serum bile acid composition in patients with atrial fibrillation. Heart 2013;99(22):1685–1692. 10.1136/heartjnl-2013-304163 23894089

[38] Canpolat S, Kirpinar I, Deveci E, Aksoy H, Bayraktutan Z, Eren I et al Relationship of asymmetrical dimethylarginine, nitric oxide, and sustained attention during attack in patients with major depressive disorder. ScientificWorldJournal 2014;2014:624395 10.1155/2014/624395 24558318 PMC3914576

[39] Psychogios N, Hau DD, Peng J, Guo AC, Mandal R, Bouatra S et al The human serum metabolome. PLoS ONE 2011;6(2):e16957 10.1371/journal.pone.0016957 21359215 PMC3040193

[40] Williams Z, Ben-Dov IZ, Elias R, Mihailovic A, Brown M, Rosenwaks Z et al Comprehensive profiling of circulating microRNA via small RNA sequencing of cDNA libraries reveals biomarker potential and limitations. Proc Natl Acad Sci U S A 2013;110(11):4255–4260. 10.1073/pnas.1214046110 23440203 PMC3600502

[41] Pritchard CC, Kroh E, Wood B, Arroyo JD, Dougherty KJ, Miyaji MM et al Blood cell origin of circulating microRNAs: a cautionary note for cancer biomarker studies. Cancer Prev Res (Phila) 2012;5(3):492–497. 10.1158/1940-6207.CAPR-11-0370 22158052 PMC4186243

[42] Krutzfeldt J, Poy MN, Stoffel M. Strategies to determine the biological function of microRNAs. Nat Genet 2006;38 Suppl:S14–S19. 16736018 10.1038/ng1799

[43] Marciniak-Czochra A, Stiehl T, Ho A.D., Jaeger W, Wagner W. Modeling of Asymmetric Cell Division in Hematopoietic Stem Cells—Regulation of Self-Renewal is Essential for Efficient Repopulation. Stem Cells Dev 2009;18(3):377–385. 10.1089/scd.2008.0143 18752377

[44] Marciniak-Czochra A, Stiehl T, Wagner W. Modeling of replicative senescence in hematopoietic development. Aging (Albany NY) 2009;1(8):723–732. 20195386 10.18632/aging.100072 PMC2830082

[45] Walenda T, Stiehl T, Braun H, Frobel J, Ho AD, Schroeder T et al Feedback signals in myelodysplastic syndromes: increased self-renewal of the malignant clone suppresses normal hematopoiesis. PLoS Comput Biol 2014;10(4):e1003599 10.1371/journal.pcbi.1003599 24763223 PMC3998886

[46] Undi RB, Kandi R, Gutti RK. MicroRNAs as Haematopoiesis Regulators. Adv Hematol 2013;2013:695754 10.1155/2013/695754 24454381 PMC3884629

[47] Chavali S, Bruhn S, Tiemann K, Saetrom P, Barrenas F, Saito T et al MicroRNAs act complementarily to regulate disease-related mRNA modules in human diseases. RNA 2013;19(11):1552–1562. 10.1261/rna.038414.113 24062574 PMC3851722

[48] Pritchard CC, Kroh E, Wood B, Arroyo JD, Dougherty KJ, Miyaji MM et al Blood cell origin of circulating microRNAs: a cautionary note for cancer biomarker studies. Cancer Prev Res (Phila) 2012;5(3):492–497. 10.1158/1940-6207.CAPR-11-0370 22158052 PMC4186243

[49] Hsieh IS, Chang KC, Tsai YT, Ke JY, Lu PJ, Lee KH et al MicroRNA-320 suppresses the stem cell-like characteristics of prostate cancer cells by downregulating the Wnt/beta-catenin signaling pathway. Carcinogenesis 2013;34(3):530–538. 10.1093/carcin/bgs371 23188675

[50] Chotinantakul K, Prasajak P, Leeanansaksiri W. Wnt1 Accelerates an Ex Vivo Expansion of Human Cord Blood CD34(+)CD38(-) Cells. Stem Cells Int 2013;2013:909812 10.1155/2013/909812 24023545 PMC3760094

[51] Lento W, Congdon K, Voermans C, Kritzik M, Reya T. Wnt signaling in normal and malignant hematopoiesis. Cold Spring Harb Perspect Biol 2013; 5(2): pii: a008011. 10.1101/cshperspect.a008011 23378582 PMC3552513

[52] Schaar DG, Medina DJ, Moore DF, Strair RK, Ting Y. miR-320 targets transferrin receptor 1 (CD71) and inhibits cell proliferation. Exp Hematol 2009;37(2):245–255. 10.1016/j.exphem.2008.10.002 19135902

[53] Qiu B, Wei F, Sun X, Wang X, Duan B, Shi C et al Measurement of hydroxyproline in collagen with three different methods. Mol Med Rep 2014;10(2):1157–1163. 10.3892/mmr.2014.2267 24858249

[54] Hudson DM, Eyre DR. Collagen prolyl 3-hydroxylation: a major role for a minor post-translational modification? Connect Tissue Res 2013;54(4–5):245–251. 10.3109/03008207.2013.848200 23772978 PMC3995746

[55] Gaillard D, Negrel R, Lagarde M, Ailhaud G. Requirement and role of arachidonic acid in the differentiation of pre-adipose cells. Biochem J 1989;257(2):389–397. 2539084 10.1042/bj2570389 PMC1135592

[56] Gysin R, Azzi A, Visarius T. Gamma-tocopherol inhibits human cancer cell cycle progression and cell proliferation by down-regulation of cyclins. FASEB J 2002;16(14):1952–1954. 12368234 10.1096/fj.02-0362fje

[57] Gall WE, Beebe K, Lawton KA, Adam KP, Mitchell MW, Nakhle PJ et al alpha-hydroxybutyrate is an early biomarker of insulin resistance and glucose intolerance in a nondiabetic population. PLoS ONE 2010;5(5):e10883 10.1371/journal.pone.0010883 20526369 PMC2878333

[58] Fimognari C, Lenzi M, Cantelli-Forti G, Hrelia P. Apoptosis and modulation of cell cycle control by bile acids in human leukemia T cells. Ann N Y Acad Sci 2009;1171:264–269. 10.1111/j.1749-6632.2009.04710.x 19723064

[59] Blank U, Karlsson G, Karlsson S. Signaling pathways governing stem-cell fate. Blood 2008;111(2):492–503. 17914027 10.1182/blood-2007-07-075168

