# Supplementary material for: MicroRNAs and Metabolites in Serum Change after Chemotherapy: Impact on Hematopoietic Stem and Progenitor Cells
Source: PLoS One. 2015 May 29;10(5):e0128231. doi: 10.1371/journal.pone.0128231 (PMC4449031; doi:10.1371/journal.pone.0128231)
Supplement: S1 File — Fig. A, Impact of serum on HSPC proliferation and immunophenotype during co-culture with MSCs. Fig. B, Correlation of HSPC proliferation with blood parameters of serum samples. Fig. C, Detected miRNA numbers correlate with leukocyte count. Fig. D, Differential miRNA expression in serum before and after chemotherapy among patient subgroups. Fig. E, Gene ontology analysis of miRNA-320c targets. Fig. F, Inhibition of miRNA-320c activity enhances HSPC proliferation. Fig. G, Correlation of metabolite levels with patient’s leukocyte count. Fig. H, Correlation of metabolite levels with patient’s thrombocyte count. Fig. I, Correlation of metabolite levels with patient’s erythrocyte count. Fig. J, Correlation of metabolite levels with patient’s hemoglobin concentration. Fig. K, Effects of metabolites on HSPCs in co-culture with MSCs. Table A, Serum samples used as cell culture supplement. Table B, Serum samples used for miRNA profiling. Table C, Serum samples used for metabolomic profiling. Table D, Detailed patient treatment information. (PDF) [file pone.0128231.s001.pdf]

Supplemental Information S1 File

MicroRNAs and Metabolites in Serum Change after Chemotherapy: Impact on Hematopoietic Stem and Progenitor Cells

Thomas Walenda[1,#], Yvonne Diener[2,#], Edgar Jost[3], Elizabeth Morin-Kensicki[4], Tamme W. Goecke[5], Andreas Bosio[2], Björn Rath[6], Tim H. Brümmendorf[3], Ute Bissels[2], Wolfgang Wagner[1]

1) Helmholtz Institute for Biomedical Engineering, RWTH Aachen University Medical School, Aachen, Germany
2) Miltenyi Biotec GmbH, Bergisch Gladbach, Germany
3) Department for Hematology, Oncology, Hemostaseology and Stem Cell Transplantation, RWTH Aachen University Medical School, Aachen, Germany
4) Metabolon, Inc., Durham, NC 27519, USA; Current affiliation: Attain, LLC, Morrisville, NC 27560, USA
5) Department of Obstetrics and Gynecology, RWTH Aachen University Medical School, Aachen, Germany
6) Department for Orthopedics, RWTH Aachen University Medical School, Aachen, Germany
#) equal contribution

Index

Fig. A: Impact of serum on HSPC proliferation and immunophenotype during co-culture with MSCs. 2
Fig. B: Correlation of HSPC proliferation with blood parameters of serum samples. 2
Fig. C: Detected miRNA numbers correlate with leukocyte count. 3
Fig. D: Differential miRNA expression in serum before and after chemotherapy among patient subgroups. 3
Fig. E: Gene ontology analysis of miRNA-320c targets. 4
Fig. F: Inhibition of miRNA-320c activity enhances HSPC proliferation. 5
Fig. G: Correlation of metabolite levels with patient's leukocyte count. 6
Fig. H: Correlation of metabolite levels with patient's thrombocyte count. 6
Fig. I: Correlation of metabolite levels with patient's erythrocyte count. 7
Fig. J: Correlation of metabolite levels with patient's hemoglobin concentration. 7
Fig. K: Effects of metabolites on HSPCs in co-culture with MSCs. 8
Table A: Serum samples used as cell culture supplement 9
Table B: Serum samples used for miRNA profiling 10
Table C: Serum samples used for metabolomic profiling 11
Table D: Detailed patient treatment information 12

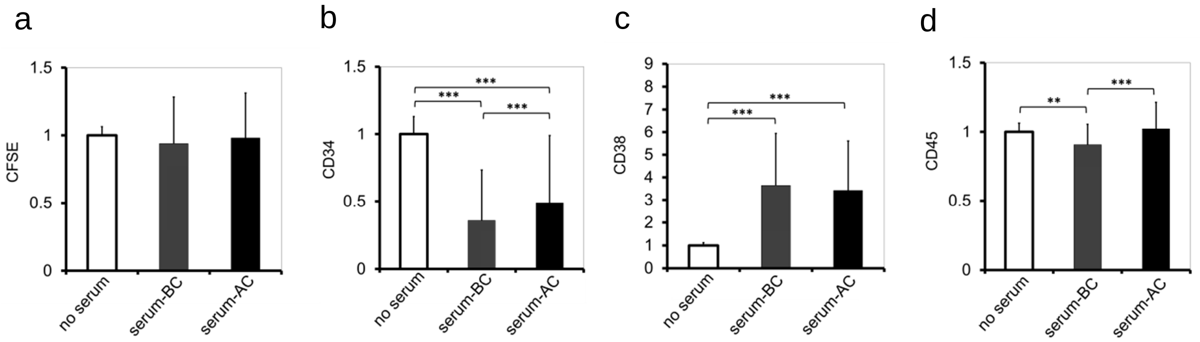

Fig. A: Impact of serum on HSPC proliferation and immunophenotype during co-culture with MSCs. HSPCs were stained with CFSE and *in vitro* co-cultured for five days with MSC feeder layers and additional serum supplements (10% serum). Serum was derived from patients before (BC) and after (AC) chemotherapy or cells were cultured in medium without serum (Ctr.). CFSE intensity (a) as well as mean fluorescence intensities of CD34 (b), CD38 (c) and CD45 (d) were then determined by flow cytometry and normalized to the intensities of corresponding control. Error bars represent SD, **P ≤ 0.01, ***P ≤ 0.001, n = 5.

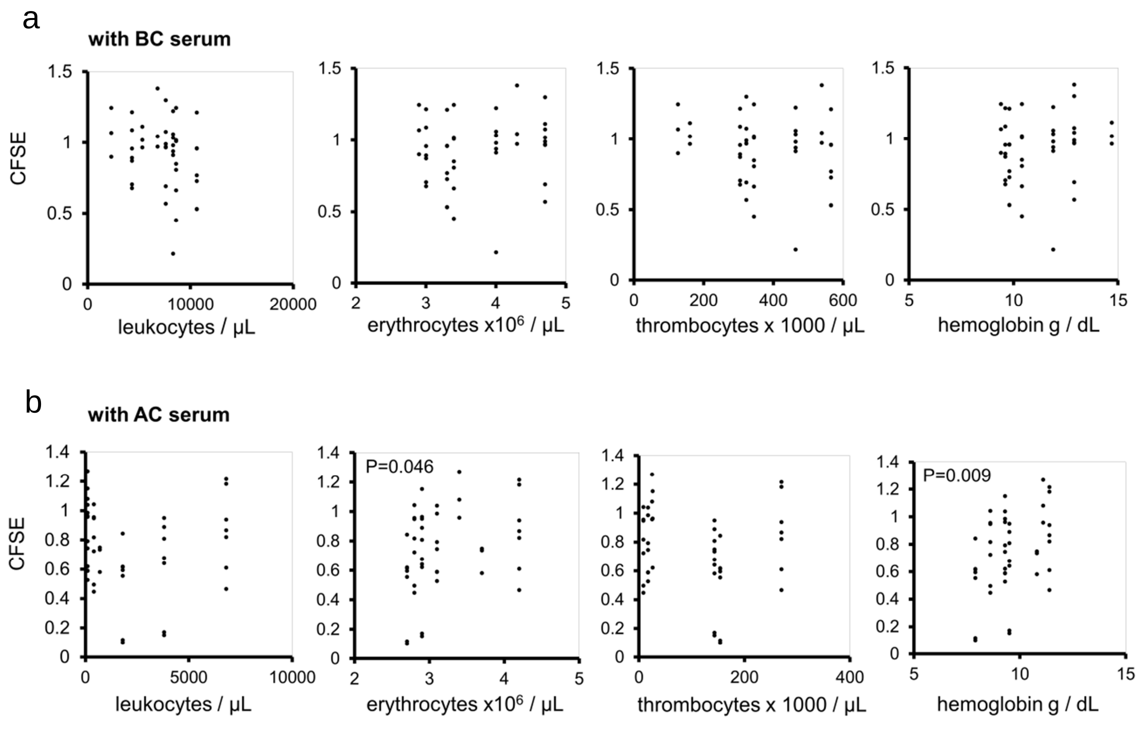

Fig. B: Correlation of HSPC proliferation with blood parameters of serum samples. Mean CFSE intensities after seven days *in vitro* culture with addition of serum samples before (a) and after therapy (b) were correlated to the corresponding blood cell counts and hemoglobin content. A significant correlation could be found for erythrocyte count and hemoglobin content with serum isolated after therapy. Pearson rank correlation revealed P-values of 0.046 and 0.009, respectively.

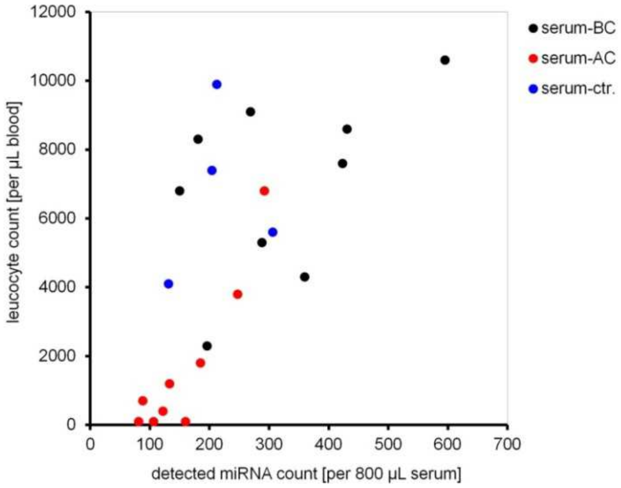

Fig. C: Detected miRNA numbers correlate with leukocyte count. The number of detected miRNAs per 800 µL of serum is plotted against the leukocyte count per µL of blood for each patient at the respective time point before (serum-BC) or and after therapy (serum-AC), as well as for healthy donors (serum-ctr.). (For two samples, blood counts were not available and the sample with a leukocyte count of 33,600 per µL was considered as outlier, Pearson correlation, P = 0.0004).

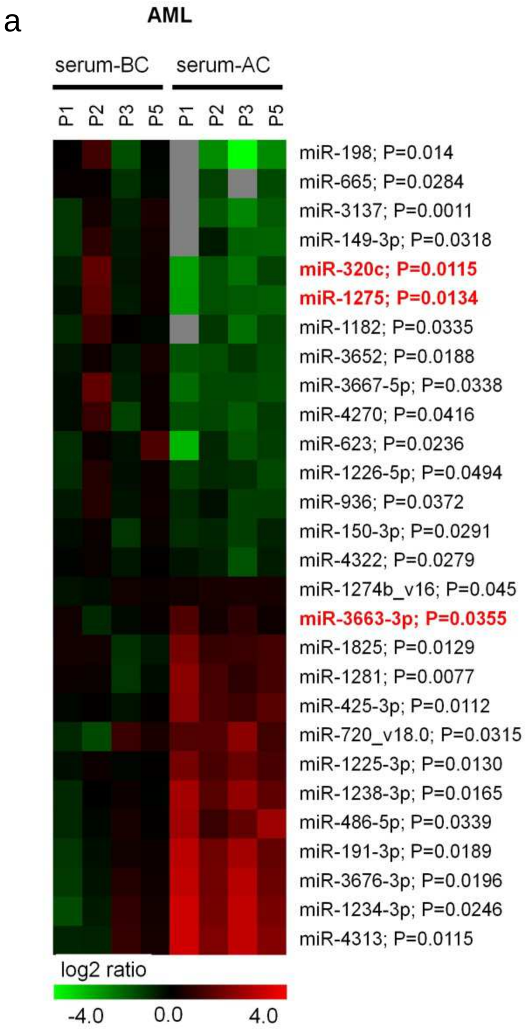

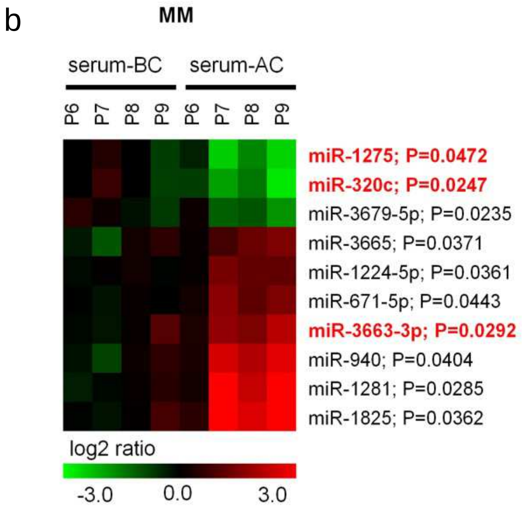

Fig. D: Differential miRNA expression in serum before and after chemotherapy among patient subgroups. (a) Heat maps of significantly differentially AML patients and (b) MM patients. Displayed is the log2-transformed ratio of the normalized signal intensities *versus* median signal intensity of BC serum. The three most significant miRNAs which overlapped in both patient groups are marked in red.

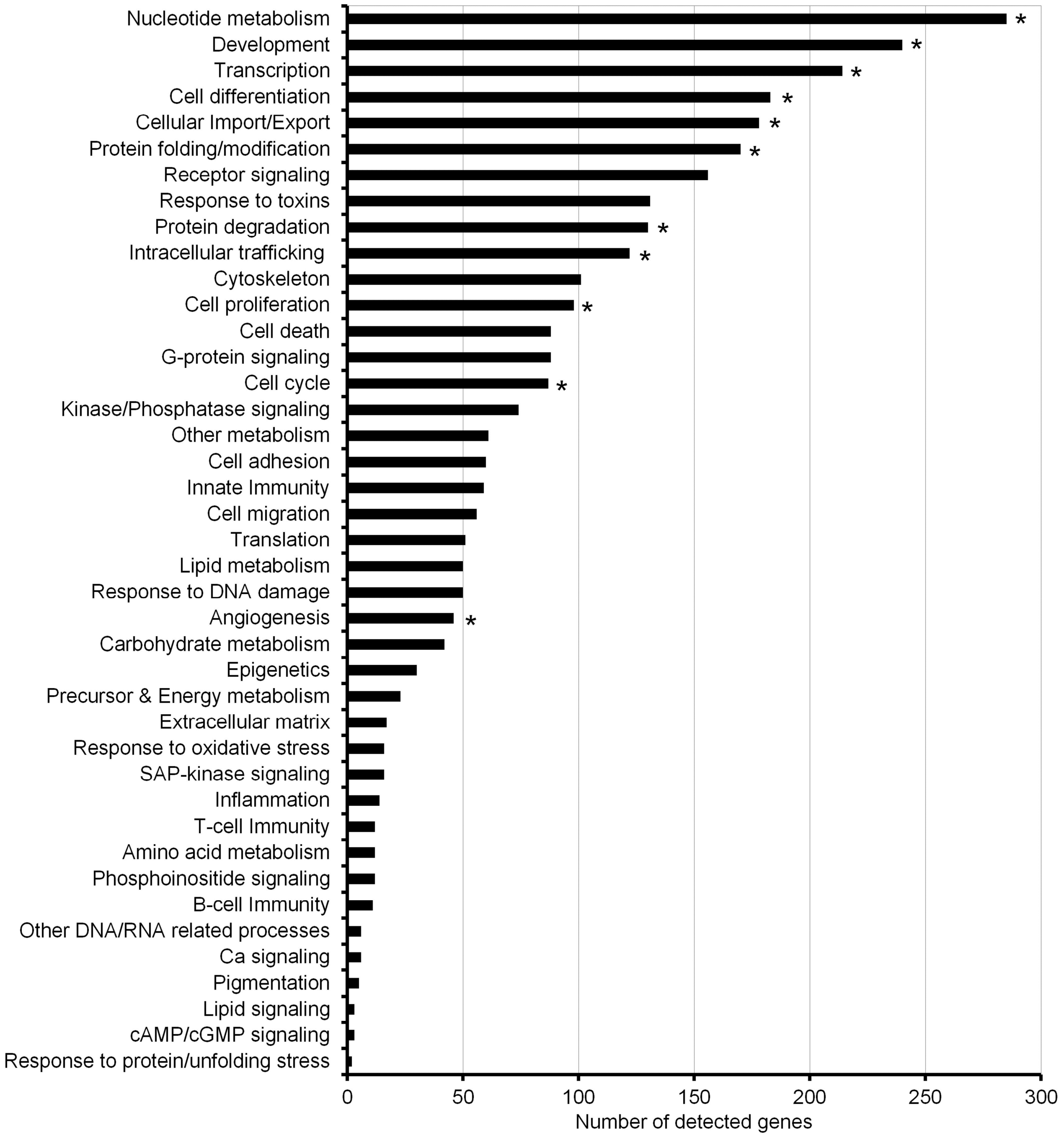

Fig. E: Gene ontology analysis of miRNA-320c targets. 768 targets of miRNA-320c were identified with by TargetScan and subjected to a term enrichment analysis based on gene ontology categories. Categories with highest probabilities (P ≤ 0.00001) according to Fisher's Exact test with Benjamini–Hochberg correction are marked with an asterisk.

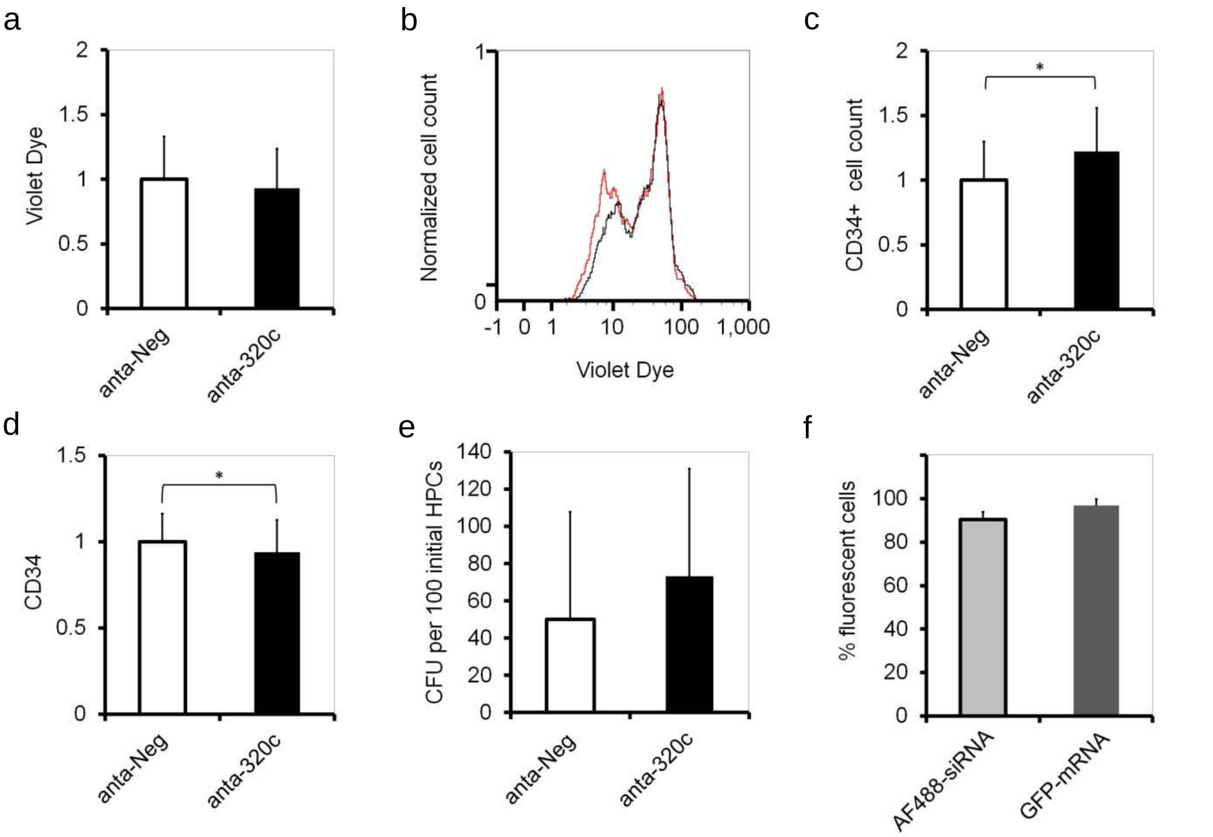

Fig. F: Inhibition of miRNA-320c activity enhances HSPC proliferation. $CD133^+$ HSPCs were electroporated with a non-targeting control antagomiR (anta-Neg) or an antagomiR targeting miRNA-320c (anta-320c). Cells were analyzed by flow cytometry four days after electroporation. (a) Violet Dye mean fluorescence intensity (MFI) normalized to control (n = 3). (b) Representative histogram of Violet Dye MFI. Black: anta-Neg, red: anta-320c. (c) $CD34^+$ cell count and (d) CD34 MFI (normalized to control, n = 4). (e) Total number of colonies after transfection with anta-320c and anta-Neg. Cells were cultured for seven days after electroporation and then re-seeded in methylcellulose medium (n = 3). (f) Efficiency of electroporation was previously determined with fluorescently labeled siRNA (AF488-siRNA, n = 3) and GFP-encoding mRNA (GFP mRNA, n = 10) – therefore we expect that the observed effects of anta-320c can be attributed to specific and sustained knockdown of miR-320c, although this was not further analyzed due to the limited number of available cells. Error bars represent SD, *$P \leq 0.05$.

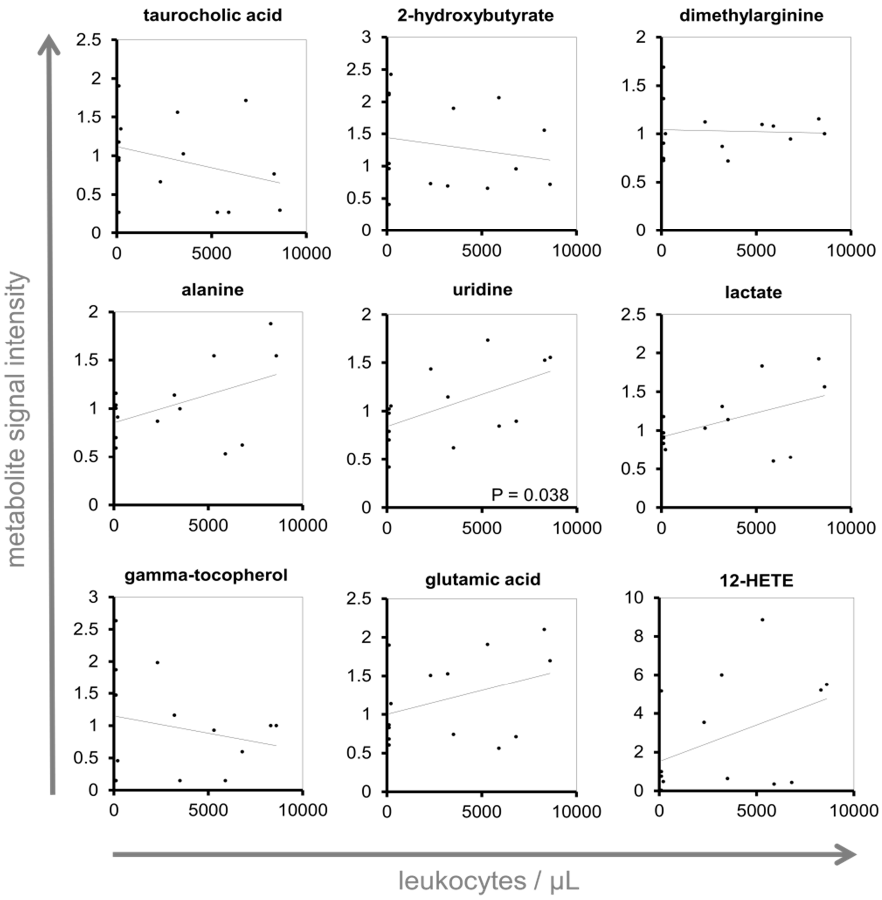

Fig. G: Correlation of metabolite levels with patient's leukocyte count. Metabolite signal intensities were correlated to the leukocyte counts. A moderate correlation could be found for uridine (P = 0.038, Pearson rank correlation).

Fig. H: Correlation of metabolite levels with patient's thrombocyte count.

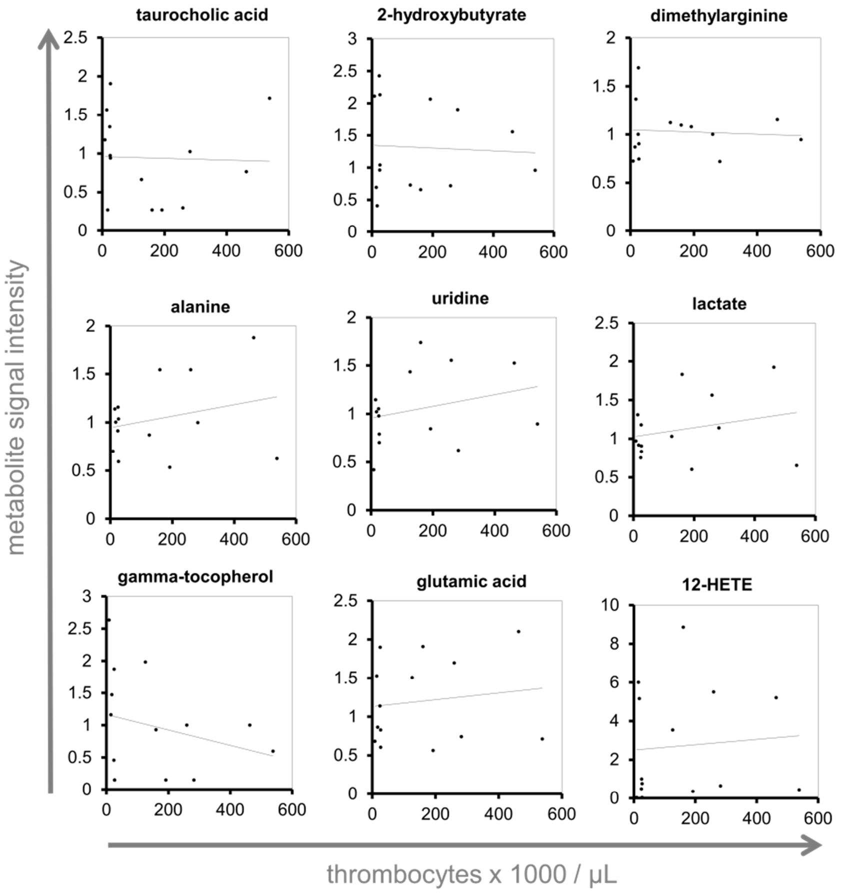

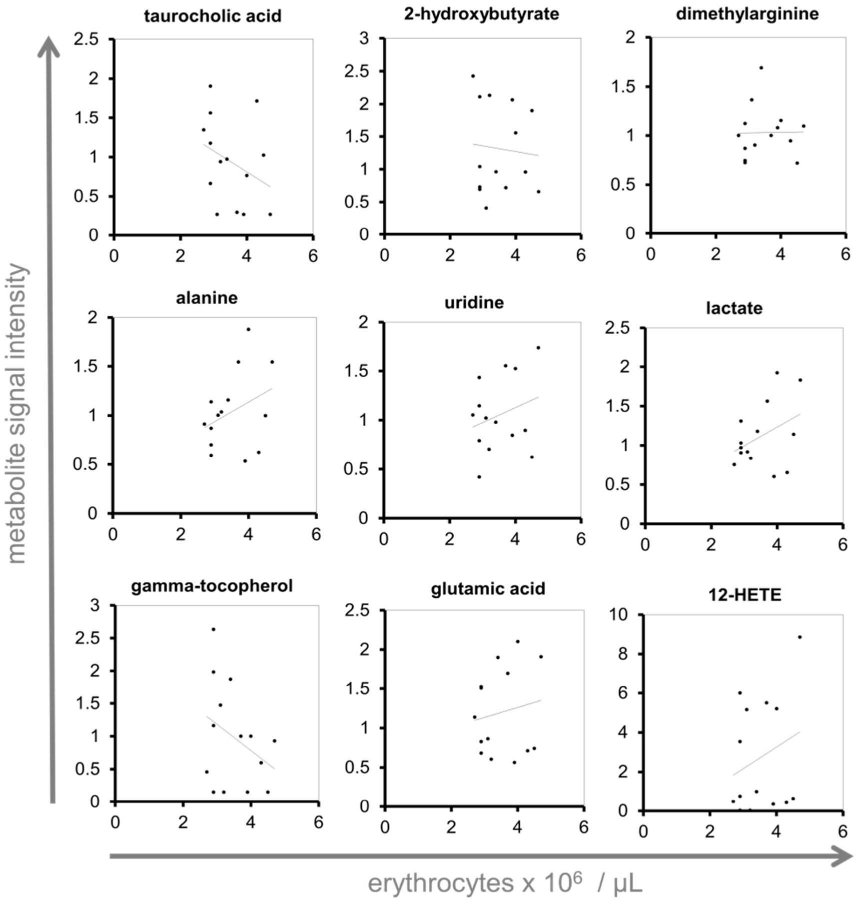

Fig. I: Correlation of metabolite levels with patient's erythrocyte count.

Fig. J: Correlation of metabolite levels with patient's hemoglobin concentration.

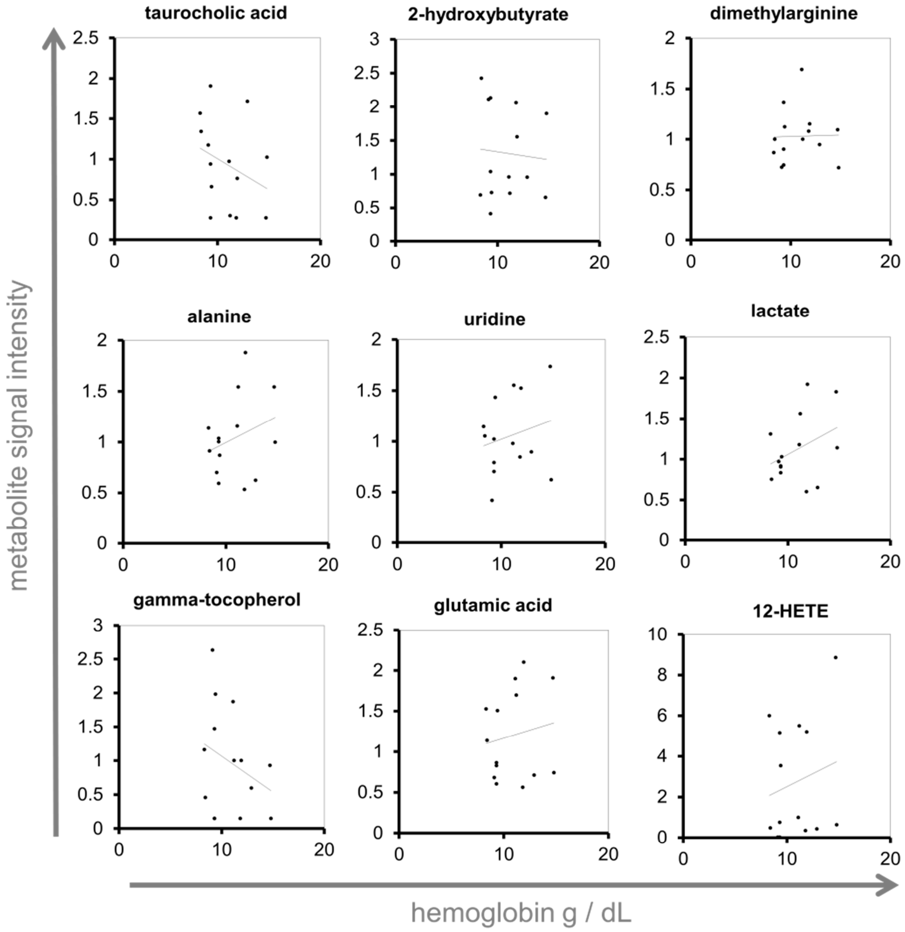

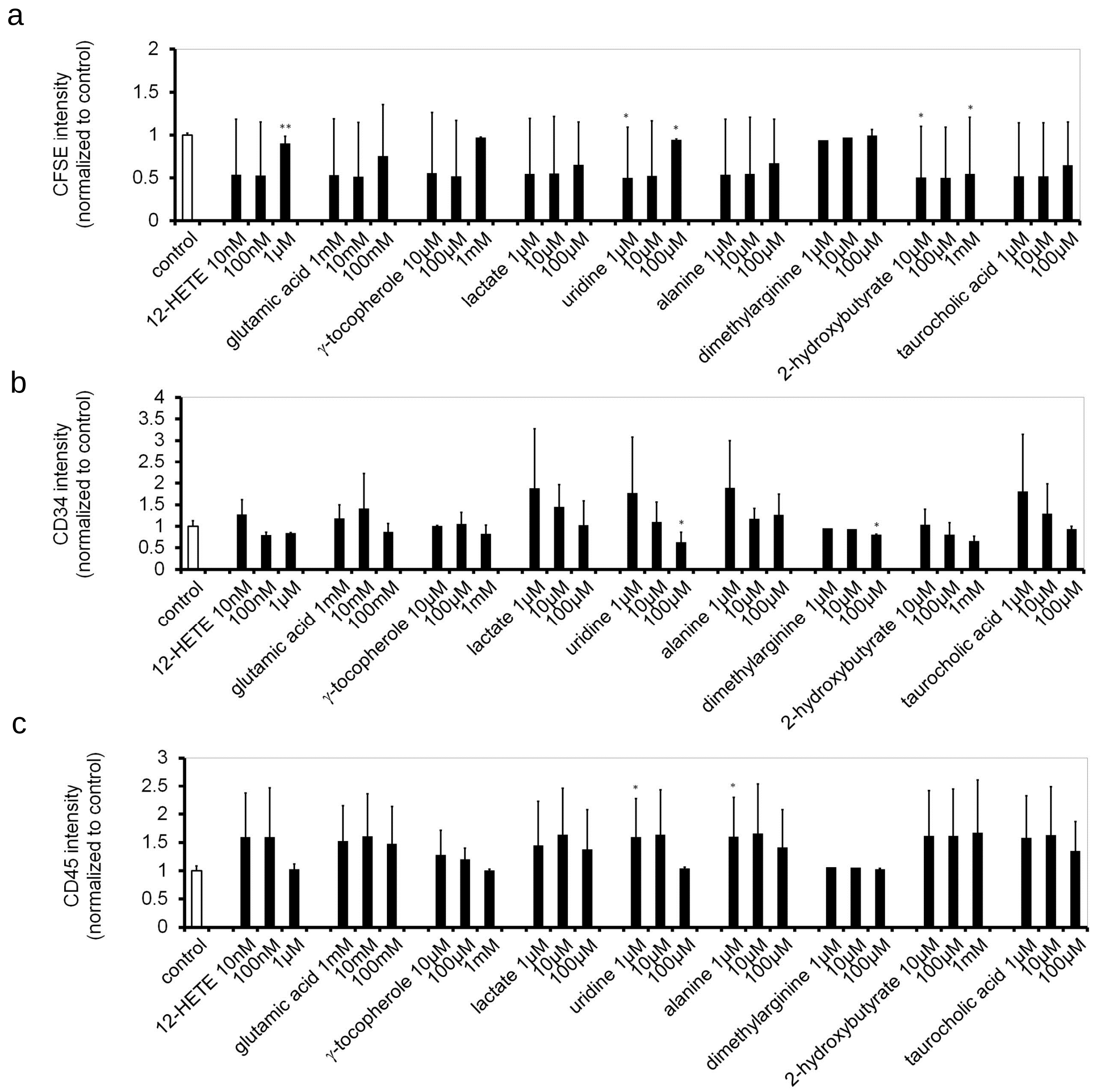

Fig. K: Effects of metabolites on HSPCs in co-culture with MSCs. HSPCs were stained with CFSE and cultured for 5 days with MSC feeder layer and metabolite as indicated. (a) CFSE intensity as well as (b) expression of CD34 and (c) CD45 were then determined via flow cytometry and normalized to the control without additional metabolites. n = 3, error bars represent SD, *P ≤ 0.05, **P ≤ 0.01.

Table A: Serum samples used as cell culture supplement

Patient ID	BC / AC	sample code ID	sex	age	Diagnosis	leukocytes [/µL]	erythroc. [1x10^6/µL]	thromboc. [x1000/µL]	HB [g/dL]
P1	BC	ACEJ-12	f	44	AML t(8;21)	8600	3.4	344	10.4
P1	AC	ACEJ-13	f	44	AML t(8;21)	400	2.8	8.6	8.6
P2	BC	ACEJ-18	m	60	AML FLT3 ITD	10600	3.3	565	9.8
P2	AC	ACEJ-19	m	60	AML FLT3 ITD	1800	2.7	154	7.9
P3	BC	ACEJ-20	m	37	AML NPM mut	9100	2.2	206	7.7
P3	AC	ACEJ-21	m	37	AML NPM mut	1200	2.8	68	9.1
P4	BC	ACEJ-22	m	47	DLBCL	7600	4.7	322	12.9
P4	AC	ACEJ-23	m	47	DLBCL	6800	4.2	270	11.4
P5	BC	ACEJ-24	f	44	AML t(8;21)	4300	3	304	9.6
P5	AC	ACEJ-25	f	44	AML t(8;21)	3800	2.9	143	9.5
P6	BC	L15	f	56	MM	8300	4	463	11.9
P6	AC	L18	f	56	MM	100	3.1	17	9.3
P7	BC	L22	m	64	MM	5300	4.7	160	14.7
P7	AC	L25	m	64	MM	100	3.4	25	11.1
H1	Ctr.	G1	f	21		9900	4.8	253	14.7
H2	Ctr.	G2	f	74		5600	4.7	199	14.3
H3	Ctr.	G9	f	23		N.D.	N.D.	N.D.	N.D.
H4	Ctr.	G10	f	23		N.D.	N.D.	N.D.	N.D.

P = patient; H = healthy donor; BC = before chemotherapy; AC = after chemotherapy; AML = acute myeloid leukemia; MM = multiple myeloma; DLBCL = diffuse large B-cell lymphoma; FLT3 = Fms-like tyrosine kinase 3; IDT = internal tandem duplication; NPM = Nucleophosmin; t = transition; N.D. not determined.

Table B: Serum samples used for miRNA profiling

Patient ID	BC / AC	sample code ID	sex	age	Diagnosis	leukocytes [/µL]	erythroc. [1x10^6/µL]	thromboc. [x1000/µL]	HB [g/dL]
P1	BC	ACEJ-12	f	44	AML t(8;21)	8600	3.4	344	10.4
P1	AC	ACEJ-13	f	44	AML t(8;21)	400	2.8	8.6	8.6
P2	BC	ACEJ-18	m	60	AML FLT3 ITD	10600	3.3	565	9.8
P2	AC	ACEJ-19	m	60	AML FLT3 ITD	1800	2.7	154	7.9
P3	BC	ACEJ-20	m	37	AML NPM mut	9100	2.2	206	7.7
P3	AC	ACEJ-21	m	37	AML NPM mut	1200	2.8	68	9.1
P4	BC	ACEJ-22	m	47	DLBCL	7600	4.7	322	12.9
P4	AC	ACEJ-23	m	47	DLBCL	6800	4.2	270	11.4
P5	BC	ACEJ-24	f	44	AML t(8;21)	4300	3	304	9.6
P5	AC	ACEJ-25	f	44	AML t(8;21)	3800	2.9	143	9.5
P6	BC	L15	f	56	MM	8300	4	463	11.9
P6	AC	L18	f	56	MM	100	3.1	17	9.3
P7	BC	L22	m	64	MM	5300	4.7	160	14.7
P7	AC	L25	m	64	MM	100	3.4	25	11.1
P8	BC	L27	m	54	MM	2300	2.9	126	9.4
P8	AC	L30	m	54	MM	100	2.9	26	9.3
P9	BC	L42	f	64	MM	6800	4.3	538	12.9
P9	AC	L45	f	64	MM	100	3.2	26	9.3
H1	Ctr.	G1	f	21		9900	4.8	253	14.7
H2	Ctr.	G2	f	74		5600	4.7	199	14.3
H3	Ctr.	G9	f	23		N.D.	N.D.	N.D.	N.D.
H4	Ctr.	G10	f	23		N.D.	N.D.	N.D.	N.D.
H5	Ctr.	G3	f	68		4100	4.5	313	14.4
H6	Ctr.	G11	f	51		N.D.	N.D.	N.D.	N.D.
H7	Ctr.	G12	f	43		7400	5	279	14.8

P = patient; H = healthy donor; BC = before chemotherapy; AC = after chemotherapy; AML = acute myeloid leukemia; MM = multiple myeloma; DLBCL = diffuse large B-cell lymphoma; FLT3 = Fms-like tyrosine kinase 3; IDT = internal tandem duplication; NPM = Nucleophosmin; t = transition; N.D. not determined.

Table C: Serum samples used for metabolomic profiling

Patient ID	BC / AC	sample code ID	sex	age	Diagnosis	leukocytes [/µL]	erythroc. [1x10^6/µL]	thromboc. [x1000/µL]	HB [g/dL]
P10	BC	L-10	m	59	NHL follicular	8600	3.7	259	11.2
P10	AC	L-13	m	59	NHL follicular	3200	2.9	14	8.3
P6	BC	L-15	f	56	MM	8300	4	463	11.9
P6	AC	L-18	f	56	MM	100	3.1	17	9.3
P7	BC	L-22	m	64	MM	5300	4.7	160	14.7
P7	AC	L-25	m	64	MM	100	3.4	25	11.1
P8	BC	L-27	m	54	MM	2300	2.9	126	9.4
P8	AC	L-30	m	54	MM	100	2.9	26	9.3
P11	BC	L-33	m	32	NHL composite	3500	4.5	282	14.8
P11	AC	L-38	m	32	NHL composite	200	2.7	24	8.4
P9	BC	L-42	f	64	MM	6800	4.3	538	12.9
P9	AC	L-45	f	64	MM	100	3.2	26	9.3
P12	BC	L-47	f	60	MM	5900	3.9	192	11.8
P12	AC	L-50	f	60	MM	<100	2.9	8	9.1

P = patient; H = healthy donor; BC = before chemotherapy; AC = after chemotherapy; AML = acute myeloid leukemia; MM = multiple myeloma; NHL = non-Hodgkin lymphoma; N.D. not determined

Table D: Detailed patient treatment information

Patient ID	sex	age	Diagnosis	Stage	Therapy at sampling	line of therapy
P1 *	f	44	AML t(8;21)	N.D.	1st cycle of consolidation chemotherapy AraC after 2 cycles of induction	1st line
P2	m	60	AML FLT3 IDT	N.D.	1st cycle of consolidation chemotherapy AraC after 2 cycles of induction	1st line
P3	m	37	AML NPM mut.	N.D.	1st cycle of induction chemotherapy AraC / Danuorubicin	1st line
P4	m	47	DLBCL	IV	chemotherapy with BEAM in refractory situation, multiple previous chemotherapies	refractory, 3rd line
P5 *	f	44	AML t(8;21)	N.D.	2nd cycle of consolidation chemotherapy AraC after 2 cycles of induction	1st line
P6	f	56	MM	III	1st autologous transplantation with high-dose Melphalan (200mg/cm^2)	1st line
P7	m	64	MM	III	2nd autologous transplantation with high-dose Melphalan (200mg/cm^2)	1st line
P8	m	54	MM	III	1st autologous transplantation with high-dose Melphalan (200mg/cm^2)	1st line
P9	f	64	MM	III	1st autologous transplantation with high-dose Melphalan (200mg/cm^2)	1st line
P10	m	59	NHL follicular	IV	2nd autologous transplantation after BEAM chemotherapy after multiple previous chemotherapies	3rd line, 1st transpl. in 2nd line
P11	m	32	NHL composite	IV	2nd autologous transplantation after BEAM chemotherapy after R-CHOP and after R-DHAP	2nd line
P12	f	60	MM	III	1st autologous transplantation with high-dose Melphalan (200mg/cm^2)	1st line

P = patient; AML = acute myeloid leukemia; MM = multiple myeloma; DLBCL = diffuse large B-cell lymphoma; NHL = non-Hodgkin lymphoma; FLT3 = Fms-like tyrosine kinase 3; IDT = internal tandem duplication; NPM = Nucleophosmin; t = transition; AraC = Cytarabine; BEAM = chemotherapy with application of Carmustine, Etoposide, AraC and Melphalan; R-CHOP = chemotherapy with application of Rituximab, Cyclophosphamide, Hydroxydaunorubicin, Oncovin and Prednisone; N.D. not determined; * identical patient.
